# Photochemically assisted synthesis of phenacenes fluorinated at the terminal benzene rings and their electronic spectra

**DOI:** 10.3762/bjoc.21.53

**Published:** 2025-03-24

**Authors:** Yuuki Ishii, Minoru Yamaji, Fumito Tani, Kenta Goto, Yoshihiro Kubozono, Hideki Okamoto

**Affiliations:** 1 Graduate School of Environmental, Life, Natural Science and Technology, Okayama University, Okayama 700-8530, Japan; 2 Division of Molecular Science, Graduate School of Science and Engineering, Gunma University, Ota, Gunma 373-0057, Japan; 3 Institute for Materials Chemistry and Engineering, Kyushu University, Fukuoka 819-0395, Japan; 4 Research Institute for Interdisciplinary Science, Okayama University, Okayama 700-8530, Japan; 5 Department of Chemistry, Faculty of Environmental, Life, Natural Science and Technology, Okayama University, Okayama 700-8530, Japan

**Keywords:** fluorescence, fluorinated aromatics, phenacene, photoreaction

## Abstract

[*n*]Phenacenes ([*n*] = 5–7), octafluorinated at the terminal benzene rings (F_8_-phenacenes: **F_8_PIC**, **F_8_FUL**, and **F_8_7PHEN**), were photochemically synthesized, and their electronic spectra were investigated to reveal the effects of the fluorination on the electronic features of phenacene molecules. F_8_-Phenacenes were conveniently synthesized by the Mallory photoreaction of the corresponding fluorinated diarylethenes as the key step. Upon fluorination on the phenacene cores, the absorption and fluorescence bands of the F_8_-phenacenes in CHCl_3_ systematically red-shifted by ca. 3–5 nm compared to those of the corresponding parent phenacenes. The vibrational progressions of the absorption and fluorescence bands were little affected by the fluorination in the solution phase. In the solid state, the absorption band of F_8_-phenacenes appeared in the similar wavelength region for the corresponding parent phenacenes whereas their fluorescence bands markedly red-shifted and broadened. These observations suggest that the intermolecular interactions of excited-state F_8_-phenacene molecules are significantly different from those of the corresponding parent molecules, most likely due to different crystalline packing motifs.

## Introduction

Polycyclic aromatic hydrocarbons (PAHs) have been subject of continuous interest not only from aspects of fundamental synthetic, structural, and physical chemistry, but also for their application in materials science, in particular, in organic electronics [1–4]. Among the structural classifications of PAHs, the representatives are acenes and phenacenes; the former consisting of linearly fused benzene-ring arrays while the latter exhibit zigzag ones due to the angular annelation. These series are known as one-dimensional graphene ribbons. As has been commonly recognized, acene molecules have been intensively and extensively studied in the organic functional materials field [5–7]. By contrast, phenacenes had been hardly applied as functional molecules in spite of their long history; phenacenes were recognized as contents in petroleum-industry residues as early as in the 19th century [8–9]. In the last decade, phenacenes were demonstrated to serve as platform materials namely in organic electronics, such as chromophores for photovoltaics [10–12], luminophores in light-emitting devices [13–15], organic semiconductors [16–18], and even as aromatic superconductors [19]. Later, parent and chemically modified phenacenes were applied to active layers in high-performance organic field-effect transistors. Thus, the phenacene molecules displayed high hole mobility [20–26] and imide-fused phenacenes served as n-type organic semiconductors [27]. It was also disclosed that donor–acceptor-type phenacenes provided environment-dependent fluorophores showing solvatochromic fluorescence behavior [28–29]. Because phenacene molecules are quite robust against an oxidative environment even under photoillumination, they are considered to be promising platforms for constructing practical organic functional molecules.

Recently, fluorinated PAHs attracted considerable attention because the introduction of fluorine atoms significantly affects their electronic features as well as molecular and crystalline structures [30–32]. For example, the fluorination of oligoacene frameworks manipulates their electronic properties as well as their solid-state packing motifs [33–36]. The most pronounced example is that pentacene serves as a p-channel organic semiconductor [37], whereas perfluoropentacene can be used as an n-channel material [38]. It has been demonstrated that the molecular structure of [7]helicenes was modified by fluorination, thus, the helicenes’ pitch was manipulated by terminal fluorination modes [39–40]. Also, the effects of fluorination on the chiroptical features of [*n*]helicenes were theoretically predicted [41].

In addition to the manipulation of electronic natures, partial fluorination of PAHs has been recognized to be significant for crystal design and engineering. Thus, molecular packing patterns of PAHs were altered depending on the positions and extent of fluorination on the molecules [42–44]. For phenacene molecules, mono- and difluorinated picenes were synthesized, and their molecular and crystal structures were systematically investigated [45]. Monofluorinated picenes, such as 6- and 13-fluoropicene, formed dimeric structures through intermolecular F∙∙∙H contacts and behaved as p-channel semiconductors. By contrast, little information is available about the effects of polyfluorination on the physical properties and structures of phenacenes. It is expected that polyfluorination of phenacene cores could produce a functional aromatic material alternative to fluorinated acenes.

**Figure 1:**
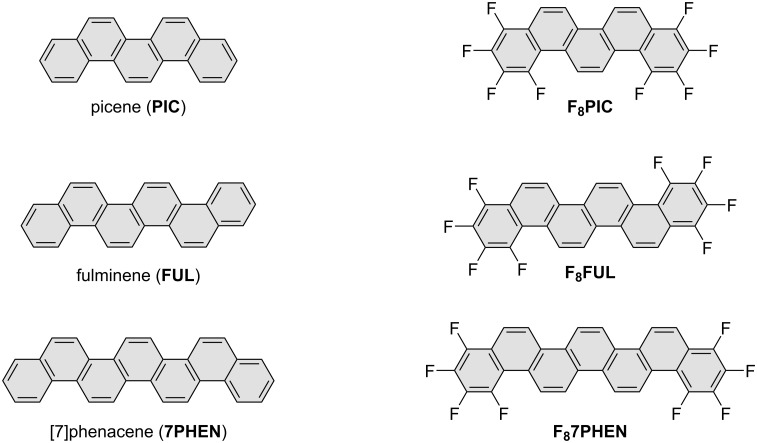
Chemical structures of phenacenes studied in this work.

In this context, it would be of interest to construct highly fluorinated phenacene derivatives and reveal their structural and electronic natures in order to develop future organic functional molecules. In this study, octafluorinated phenacenes, F_8_-phenacenes **F_8_PIC**, **F_8_FUL**, and **F_8_7PHEN** (see Figure 1 for their chemical structures), were systematically synthesized via the Mallory photoreaction [46] as the key step, and their UV–vis and fluorescence spectra were investigated in comparison with those of the corresponding parent phenacenes **PIC**, **FUL**, and **7PHEN** to reveal the effects of the fluorination at the terminal rings.

## Results and Discussion

### Synthesis of F_8_-phenacenes

**Scheme 1:**
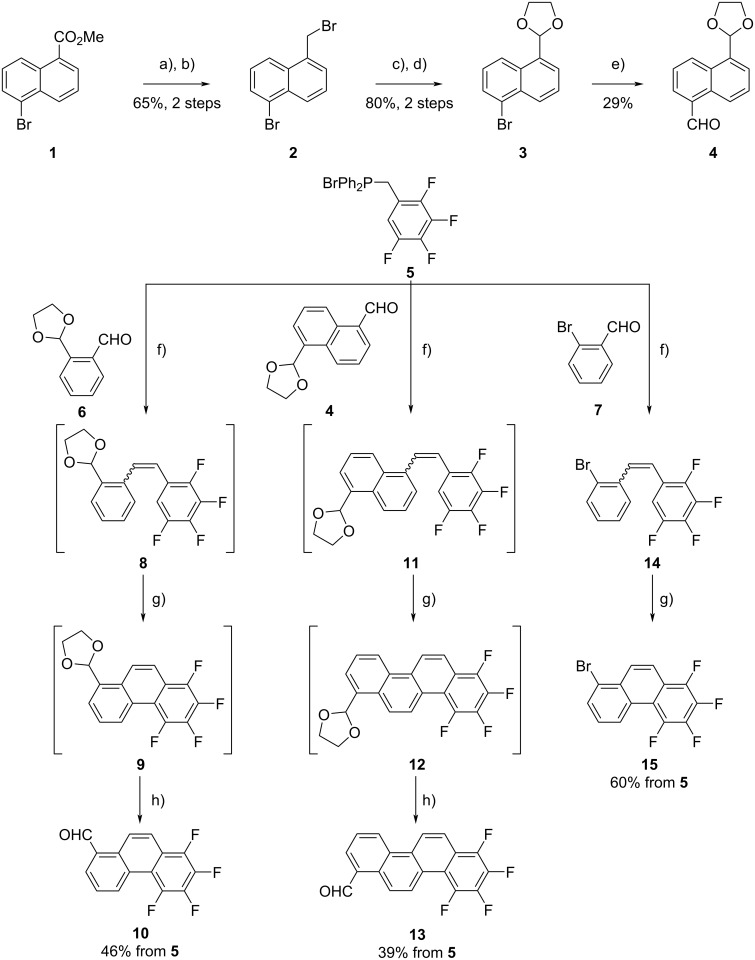
Synthesis of building blocks **10**, **13**, and **15**. Reagents and conditions: a) NaBH_4_, MeOH, THF, reflux; b) PBr_3_, reflux; c) *N*-methylmorpholine-*N*-oxide, THF, reflux; d) ethylene glycol, *p*-TsOH, toluene, reflux; e) *tert*-BuLi, DMF, THF, −78 °C; f) KOH, 18-crown-6, CH_2_Cl_2_, reflux (rt for **8**); g) *h*ν, I_2_, cyclohexane (toluene/THF mixture for **11**); h) *p*-TsOH, acetone, reflux.

**Scheme 2:**
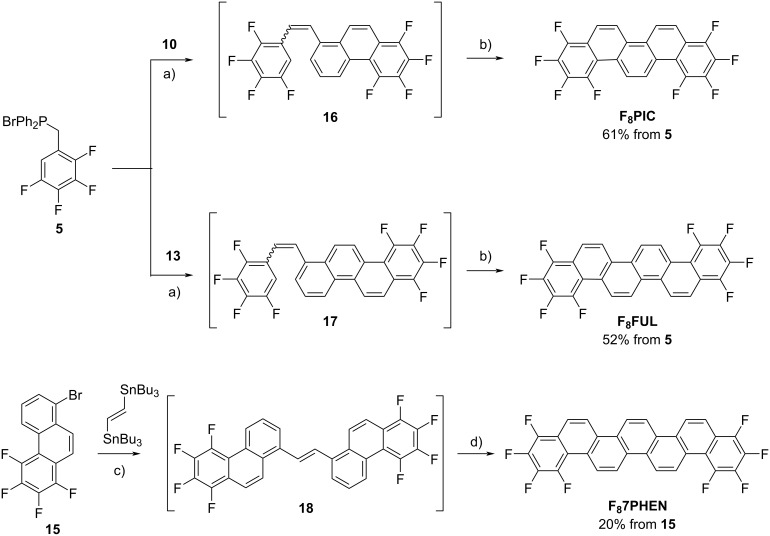
Synthesis of **F_8_PIC**, **F_8_FUL**, and **F_8_7PHEN**. Reagents and conditions: a) KOH, 18-crown-6, CH_2_Cl_2_, reflux; b) *h*ν, I_2_, toluene, rt (reflux for **F_8_FUL**); c) Pd(PPh_3_)_4_, toluene, reflux; d) *h*ν, I_2_, toluene, reflux.

The synthetic routes to building blocks **10**, **13**, and **15** and those to the desired **F_8_PIC**, **F_8_FUL**, and **F_8_7PHEN** are respectively shown in Scheme 1 and Scheme 2. The newly synthesized compounds were characterized by NMR spectroscopy as well as elemental analysis or high-resolution mass spectrometry. The experimental details and compound data are provided in Supporting Information File 1.

Aldehyde **4**, in which one of the formyl groups in napthalene-1,5-dicarboxaldehyde was protected as an acetal, was prepared through a 5-step reaction sequence. Phosphonium salt **5** [39] and the partly protected *o*-phthalaldehyde **6** [47] were obtained by previously reported procedures.

The reaction of phosphonium salt **5** with aldehyde **6** in the presence of KOH and 18-crown-6 produced fluorine-containing diarylethene **8** as a mixture of *E*- and *Z*-isomers. Subsequently, the *E*/*Z* mixture of **8** was subjected to the Mallory photoreaction without separation. Thus, compound **8** was irradiated with fluorescent black-light lamps (300 nm, 6 × 16 W) in the presence of a catalytic amount of I_2_ under an aerated condition. After the photolysis, the acetal moiety was partly cleaved to produce a mixture of acetal **9** and aldehyde **10**. The obtained reaction mixture was treated with TsOH in acetone to afford desired fluorinated phenanthrenecarbaldehyde **10** in moderate yield (46% from **5**). The homologous chrysenecarbaldehyde **13** was obtained starting from aldehyde **4** via the same two-step procedure in a 39% yield from **5**. Bromophenanthrene derivative **15** was prepared by Wittig reaction between compounds **5** and **7** followed by Mallory photoreaction in a 60% yield from **5**.

The target compounds, **F_8_PIC** and **F_8_FUL**, were obtained through the Wittig-reaction and Mallory-photoreaction sequence (Scheme 2). Thus, reactions between phosphonium salt **5** and specific aldehydes, **10** and **13**, followed by photolysis in the presence of I_2_ and O_2_, respectively, afforded **F_8_PIC** (57%) and **F_8_FUL** (52%). **F_8_7PHEN** was obtained by a Migita–Kosugi–Stille coupling between bromophenanthrene **15** and (*E*)-1,2-bis(tributylstannyl)ethene to afford diarylethene **18** followed by Mallory photoreaction. The obtained intermediate **18** contained residual palladium and isolation was not successful due to its poor solubility in common organic solvents. Therefore, the crude **18** was subjected to the Mallory photoreaction without purification. Photoirradiation of diarylethene **18** was performed in the presence of a catalytic amount of I_2_ in refluxing toluene to afford **F_8_7PHEN** which was isolated by sublimation under vacuum.

### Absorption and fluorescence spectra of F_8_-phenacenes

**Figure 2:**
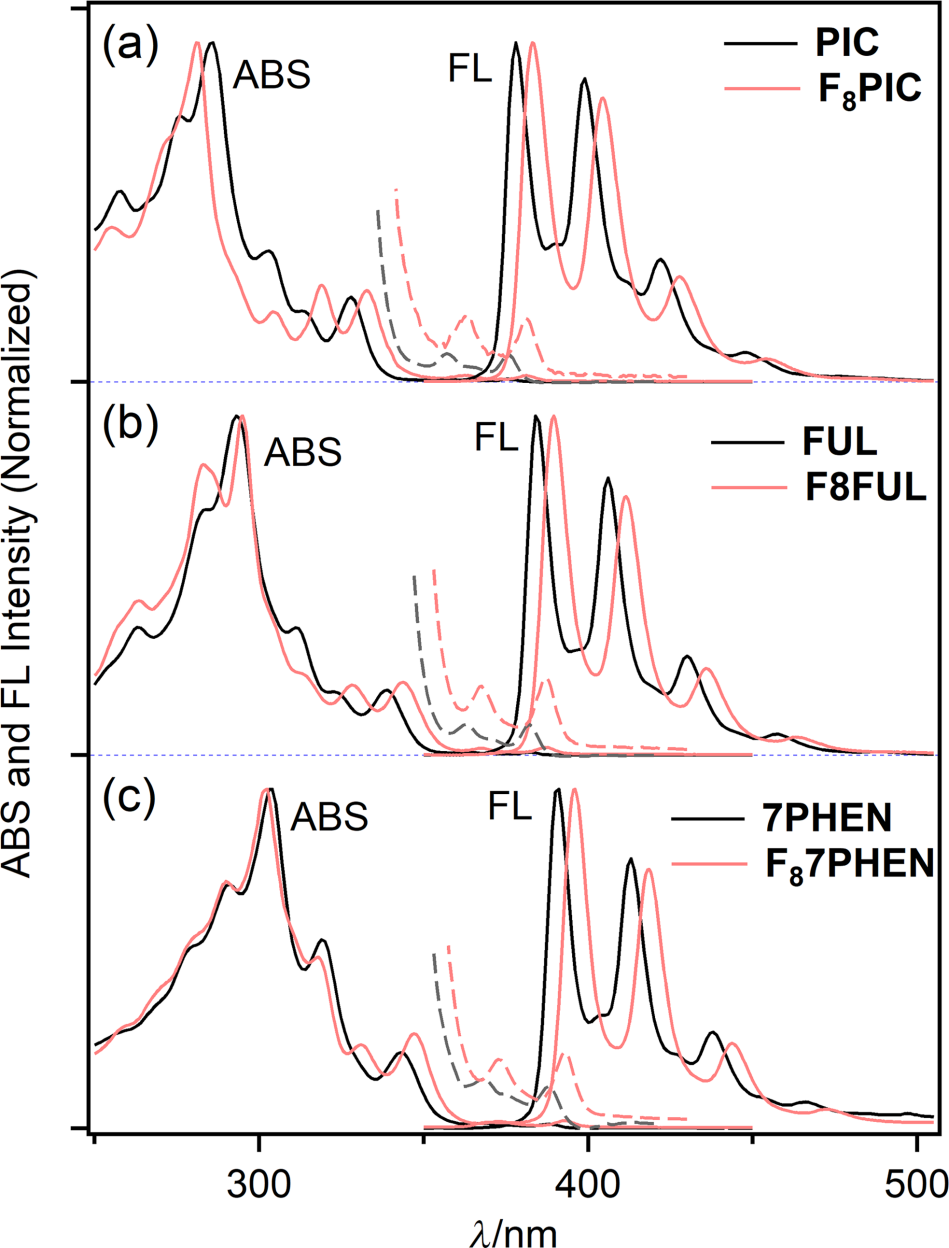
UV–vis and fluorescence spectra of **F_8_PIC** (a), **F_8_FUL** (b), and **F_8_7PHEN** (c) (red lines) and the corresponding parent phenacenes (black lines) in CHCl_3_. The broken lines show long-wavelength absorption bands at 10-times magnification of the intensity for clarity.

In order to get insights into the electronic characteristics of **F_8_PIC**, **F_8_FUL**, and **F_8_7PHEN**, their UV–vis and fluorescence spectra were measured in CHCl_3_ (Figure 2). The electronic spectra of the corresponding parent phenacenes [48–49] are also illustrated as reference. The selected photophysical parameters are summarized in Table 1. The fluorescence excitation spectra were consistent with absorption spectra to unambiguously assign the fluorescence bands to the studied F_8_-phenacenes (Figure S1 in Supporting Information File 1).

**Table 1:** Photophysical parameters for F_8_-phenacenes and the parent phenacenes.

Compound	λ_ABS_/nm		λ_FL_/nm		Δλ_FL_/nm (Δν̃_FL_/cm^−1^)^b^

	(α band)^a^	(*p* band)^a^	in CHCl_3_ (Φ_F_)	in solid	

**F** **_8_** **PIC**	381	333	383 (0.08)	458	75 (4280)
**F** **_8_** **FUL**	387	344	390 (0.12)	467	77 (4230)
**F** **_8_** **7PHEN**	393	347	396 (0.08)	489	93 (4800)
**PIC**	376^c^	328^c^	380 (0.09)^c^	391 sh, 408	28 (1810)
**FUL**	382^c^	339^c^	386 (0.12)^c^	398 sh, 416	30 (1870)
**7PHEN**	388^d^	343^d^	391 (0.12)^d^	407 sh, 424^d^	33 (1990)

^a^The Clar’s descriptions for the absorption bands; ^b^Δλ_FL_ = λ_FL_(solid) − λ_FL_(CHCl_3_), Δλ_FL_ = λ_FL_(solid) − λ_FL_(CHCl_3_), Δν̃_FL_ = ν̃_FL_ (CHCl_3_) − ν̃_FL_ (solid); ^c^Ref. [48]; ^d^Ref. [49].

In the UV–vis spectra, a small-intensity band at 376–393 nm and a moderate-intensity one at 333–347 nm were observed for all compounds studied. The former and the latter absorption bands are, respectively, assigned to the α- and *p*-bands according to Clar’s description [50]. The absorption bands only slightly red-shifted upon the π-extension (Δλ_ABS_ = ca. 6 nm per increment of one benzene ring) and the spectral profiles resemble each other irrespective of the length of the phenacene π conjugation and the fluorine substitution. The results suggest that these factors could provide insignificant effects on the apparent electronic spectral nature of the phenacenes in solution. The fluorescence spectra of the phenacenes displayed well-resolved vibrational structures as characteristics of rigid aromatic molecules. Like the UV–vis spectral behavior, the fluorescence bands gradually red-shifted with increasing the benzene-ring numbers, by ca. 6 nm per increment of one benzene ring, for both F_8_-phenacenes and the parent phenacenes.

As seen in Figure 2, it is characteristic that the α- and *p*-absorption bands and fluorescence bands systematically red-shifted by 5–6 nm compared to the parent molecules upon the fluorination. The effects of fluorination on UV–vis and fluorescence spectral behavior was similar to those reported for fluorinated [7]helicenes [38]. The fluorescence quantum yields, Φ_F_ of F_8_-phenacenes were ca. 0.1 (0.08 for **F_8_PIC**, 0.12 for **F_8_FUL**, and 0.08 for **F_8_7PHEN**) which were similar to those of the corresponding parent phenacenes [48–49]. Thus, in the solution phase, the introduction of fluorine substituents provided seemingly minimal effects on the electronic spectral features of phenacenes.

**Figure 3:**
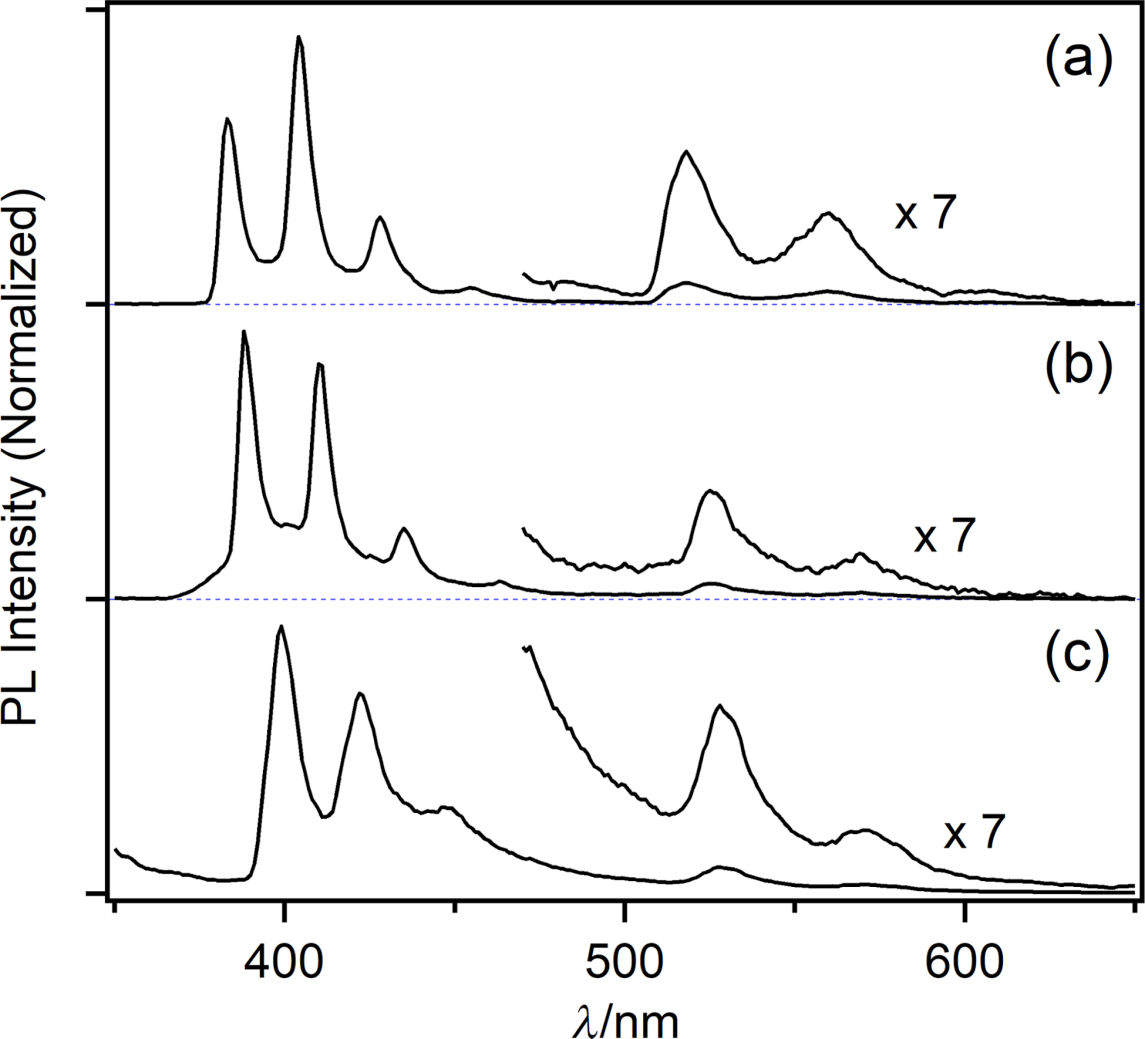
Photoluminescence spectra of **F_8_PIC** (a), **F_8_FUL** (b), and **F_8_7PHEN** (c) in toluene at 77 K.

It has been demonstrated that parent phenacenes phosphoresce in a 500–620 nm wavelength region at 77 K showing the clear vibrational progression. The first phosphorescence vibrational peak was observed at 501 nm for **PIC** and 514 nm for **FUL** [48]. In the case of F_8_-phenacenes, at 77 K, in addition to fluorescence bands, photoluminescence bands assignable to phosphorescence were detected (λ_PHOS_ = 518 nm for **F_8_PIC**, 528 nm for **F_8_PIC**, and 525 nm for **F_8_7PHEN**, Figure 3). The red-shift for phosphorescence upon the octafluorination was slightly more pronounced compared to that for fluorescence. The fact that the phosphorescence bands were observed for F_8_-phenacenes indicates that intersystem crossing is operative as one of the non-radiative processes contributing to the low fluorescence quantum yield of the F_8_-phenacenes (cf*.* Table 1).

**Figure 4:**
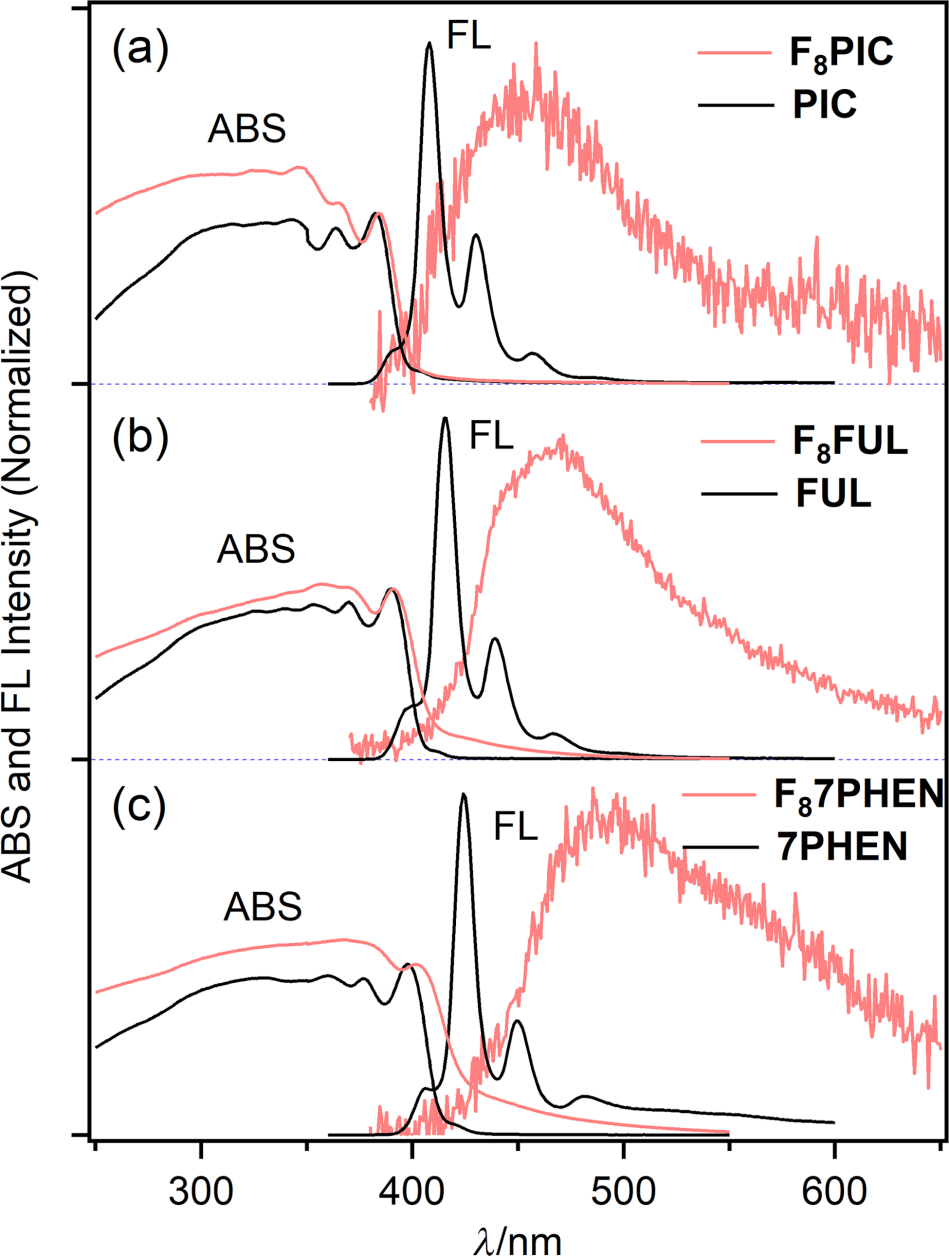
Electronic spectra of **F_8_PIC** (a), **F_8_FUL** (b), and **F_8_7PHEN** (c) (red lines) and the corresponding parent phenacenes (black lines) in the solid state.

Because fluorescence behavior in the solid state reflects molecular alignment and intermolecular interactions in the crystals, solid-state fluorescence of the F_8_-phenacenes was investigated. We observed fluorescence behavior significantly different from those observed in solution. Figure 4 compares absorption and fluorescence spectra between F_8_-phenacenes and the parent phenacenes in the solid phase.

The parent phenacenes displayed sharp and vibrationally resolved fluorescence bands with maxima in the 380–390 nm region which were consistent with those observed in solution. By contrast, for the F_8_-phenacenes, the solid-state fluorescence spectra significantly broadened and red-shifted compared to those observed in solution: **F_8_PIC**, **F_8_FUL**, and **F_8_7PHEN**, respectively, displayed fluorescence maxima at 458, 467, and 489 nm. As shown in Table 1, the differences in the fluorescence maxima shifts between the solution and solid phases (Δλ_FL_ and Δν̃_FL_) are obviously larger for F_8_-phenacenes than the parent phenacenes. These observations explicitly indicate that the alignment of the phenacene molecules in the solid phase changed upon the fluorination. It can be expected that the fluorinated phenacenes are aligned in a π–π interacting manner to display the excimeric fluorescence band in the solid state. Previously, excimer fluorescence of picene chromophores was observed in a 450–650 nm wavelength region for a macrocyclic picenophadiene [51]. As the solid-state fluorescence bands of the F_8_-phenacenes were observed in the similar wavelength region of the picenophadiene, the solid-state F_8_-phenacene molecules have excimeric character in the fluorescing state.

It is critical to know the crystal packing of the F_8_-phenacenes for clarifying the different solid-fluorescence behavior between the parent and fluorinated phenacenes. Although we have extensively examined crystallization of F_8_-phenacenes, no single crystal suitable for an X-ray diffraction analysis was obtained. The crystalline structural analysis is still under examination.

### Theoretical analyses

**Figure 5:**
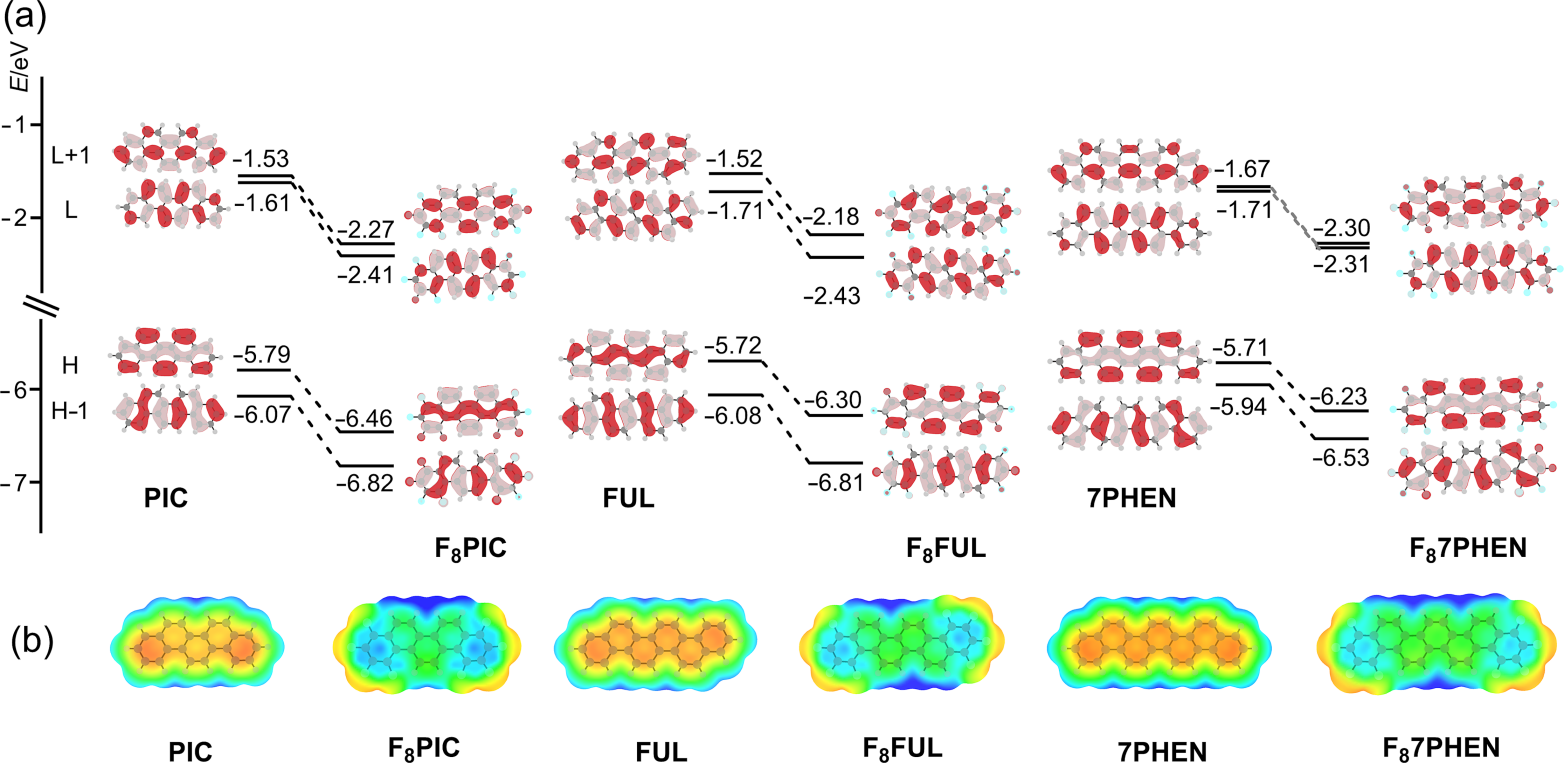
(a) The MO diagrams of the parent and fluorinated phenacenes (B3LYP/6-31+G(d,p)). H and L, respectively, denote HOMO and LUMO; (b) electrostatic potentials mapped on the total S_0_ electron density surface in the ground state. The orange and blue sites, respectively, indicate negative and positive regions (−0.02 ≈ 0.02 hartree).

Quantum chemical studies were performed to understand the electronic spectral behavior of the F_8_-phenacenes. The calculation level at B3LYP/6-31+G(d,p) [52] was chosen because the B3LYP functional qualitatively reproduced the experimental properties of some fused aromatics [53]. Figure 5 shows the molecular orbitals (MOs) around the frontier MO levels and the eigenvalues.

The shapes (symmetries) and the energy-level order of the MOs in the F_8_-phenacenes are the same as those of the corresponding parent phenacenes. It is characteristic that, upon the fluorination, the MO levels systematically lowered by 0.7–0.8 eV compared to the parent phenacenes, and that the energy gaps and the symmetry of the MOs are little affected by the fluorination. Accordingly, the electronic spectral behavior of the F_8_-phenacenes is essentially the same as that of the parent phenacenes. The theoretical calculation results were consistent with the experimentally observed electronic spectral features in solution, i.e., the absorption and fluorescence bands only slightly red-shifted (by 5–10 nm) and the spectral shapes were little affected by the fluorination. It can be concluded that, through the fluorination, one can tune the MO energy levels without changing the apparent electronic spectral features in solution, such as, electronic spectral shapes, wavelengths, and fluorescence quantum yields. Such fluorine-substitution effects on electronic spectra and MO levels were recognized for fluorinated oligoacene systems [30,34,36].

The theoretical calculations suggest that, in the ground state, the polarization of the phenacene molecules inverted upon the introduction of fluorine atoms. As seen from electrostatic potential (ESP) mapping (Figure 5b), the phenacene cores are negative (orange region) in the parent compounds, whereas the phenacene cores turned to be positive (blue) for the F_8_-phenacenes. Therefore, one can manipulate the polarization of the phenacene cores through the introduction of fluorine atoms without altering the electronic spectral features.

The excited-state electronic characteristics were also calculated, and the electronic transitions for the first two vertical absorption bands are summarized in Table 2. Although the calculated S_1_←S_0_ and S_2_←S_0_ electronic transition energies were slightly overestimated to show a systematic difference between the experimental and calculated absorption wavelengths, Δλ_1_ = 21–26 nm for the S_1_←S_0_ transition and Δλ_2_ = 11–14 nm for the S_2_←S_0_ transition, the calculation results qualitatively explain the absorption spectral behavior of the fluorinated phenacenes (cf. Table S2 in Supporting Information File 1). Therefore, the S_0_–S_1_ transition band, experimentally observed in the 381–393 nm wavelength region for F_8_-phenacenes, was assigned to a forbidden transition contributed from (H − 1)-to-L and H-to-(L + 1) electronic transitions (α band according to Clar’s description with H and L, respectively, denote HOMO and LUMO). The S_2_←S_0_ transition is assignable to H–L one (*p*-band). The calculated results, in particular for **F_8_PIC** and **F_8_7Phen** possessing odd benzene-ring π conjugations, were well consistent with the electronic transition characteristics of phenacenes [54]. In the case of **F_8_FUL** possessing an even-benzene-ring homologue structure, there was a significant contribution from the H–L transition in the α band, presumably because of the difference in the molecular symmetry.

**Table 2:** Calculated vertical electronic transitions for F_8_-phenacenes and the parent phenacenes.

Compound	S_1_←S_0_ (α band)				S_2_←S_0_ (*p* band)			

	λ_1_/nm^a^	*f* ^b^	configuration^c^		λ_2_/nm^a^	*f* ^b^	configuration^c^	

**F** **_8_** **PIC**	355	0.0109	H − 1→L H→L + 1	(28%) (68%)	344	0.2276	H − 1→L + 1 H→L	(10%) (90%)

**F** **_8_** **FUL**	365	0.0731	H − 1→L H→L H→L + 1	(12%) (62%) (19%)	356	0.1742	H − 1→L H→L H→L + 1	(18%) (31%) (51%)

**F** **_8_** **7PHEN**	372	0.0160	H − 1→L H→L + 1	(18%) (73%)	361	0.3166	H→L	(90%)

**PIC**	350	0.0053	H − 1→L H→L + 1	(35%) (65%)	336	0.1602	H − 1→L + 1 H→L	(14%) (86%)

**FUL**	357	0.0162	H − 1→L H→L H→L + 1	(29%) (22%) (46%)	349	0.1768	H→L H→L + 1	(68%) (19%)

**7PHEN**	366	0.0085	H − 1→L H→L + 1	(28%) (65%)	355	0.2497	H − 1→L + 1 H→L	(11%) (88%)

^a^Calculated wavelengths of vertical transitions for S_1_←S_0_ (λ_1_) and S_0_←S_2_ (λ_2_); ^b^calculated oscillator strength; ^c^configurations for the electronic transitions, with H and L, respectively, denote HOMO and LUMO. Electronic transitions with low contribution (<10%) are omitted.

## Conclusion

Octafluorinated phenacenes, **F_8_PIC**, **F_8_FUL**, and **F_8_7PHEN**, were conveniently synthesized through the Mallory photoreaction as the key step, and their electronic spectral features were investigated. They displayed UV–vis and fluorescence spectra which were seemingly the same as those of the parent phenacene molecules in the solution phase although theoretical calculations predicted the MO energy levels of F_8_-phenacenes markedly lowered by the fluorination. By contrast, in the solid phase, **F_8_PIC**, **F_8_FUL**, and **F_8_7PHEN** showed significantly broadened and red-shifted fluorescence bands indicating that the intermolecular interactions in the solid phase were modified by the fluorine substitution. The present results could provide a strategy for the manipulation of the solid-state optoelectronic nature of polycyclic aromatic molecules to develop future functional materials in organic electronics.

## Supporting Information

File 1 Excitation spectra of the fluorescence, synthetic procedures and physical data for the new compounds, theoretical calculation results, copy of ^1^H and ^13^C NMR spectra of the prepared compounds.

## Data Availability

All data that supports the findings of this study is available in the published article and/or the supporting information of this article.
