# Supplementary material for: Photochemically assisted synthesis of phenacenes fluorinated at the terminal benzene rings and their electronic spectra
Source: Beilstein J Org Chem. 2025 Mar 24;21:670–9. doi: 10.3762/bjoc.21.53 (PMC11956079; doi:10.3762/bjoc.21.53)
Supplement: File 1 — Excitation spectra of the fluorescence, synthetic procedures and physical data for the new compounds, theoretical calculation results, copy of 1H and 13C NMR spectra of the prepared compounds. [file Beilstein_J_Org_Chem-21-670-s001.pdf]

BEILSTEIN JOURNAL OF ORGANIC CHEMISTRY

Supporting Information

for

Photochemically assisted synthesis of phenacenes fluorinated at the terminal benzene rings and their electronic spectra

Yuuki Ishii, Minoru Yamaji, Fumito Tani, Kenta Goto, Yoshihiro Kubozono and Hideki Okamoto

Beilstein J. Org. Chem. **2025,** *21,* 670–679. doi:10.3762/bjoc.21.53

Excitation spectra of the fluorescence, synthetic procedures and physical data for the new compounds, theoretical calculation results, copy of ^{1}H and ^{13}C NMR spectra of the prepared compounds

License and Terms: This is a supporting information file under the terms of the Creative Commons Attribution License (https://creativecommons.org/licenses/by/4.0). Please note that the reuse, redistribution and reproduction in particular requires that the author(s) and source are credited and that individual graphics may be subject to special legal provisions.
The license is subject to the *Beilstein Journal of Organic Chemistry* terms and conditions: (https://www.beilstein-journals.org/bjoc/terms)

Table of contents

1. Experimental S2–S15
2. Fluorescence excitation spectra S16
3. Theoretical calculation results S17–S23
4. NMR spectra of the synthesized compounds S24–S51
5. References S52–S53

1. Experimental

1-1. General aspects

^1H, ^{19}F and ^{13}C NMR spectra were recorded on VARIAN NMR System 600 (600 MHz) and JEOL JNM-ECZ600R (600 MHz) spectrometers. Hexafluorobenzene (C_6F_6) was used as the internal standard for the measurement of ^{19}F NMR spectra [S1].

Absorption spectra were recorded on a JASCO V-530 and a SHIMADZU UV-1850 spectrophotometers. Solid-state absorption spectra were obtained with JASCO V-750 spectrophotometer. Corrected fluorescence spectra were obtained with JASCO 5300 and HITACHI F2500 spectrophotometers. Fluorescence quantum yields were determined using an absolute photoluminescence (PL) quantum yield measurement system (C13534-01 from HAMAMATSU PHOTONICS K.K.).

Elemental analyses were obtained using Perkin-Elmer 2400II CHN-S analyzer at the Micro Elemental Analysis Laboratory of Okayama University. High-resolution mass spectra (FAB) were recorded on a JEOL JMS-700 MStation spectrometer at the Institute for Materials Chemistry and Engineering, Kyushu University.

1-2. Synthesis of the building blocks

1-2-1. Synthesis of 5-(1,3-dioxolan-2-yl)-1-naphthaldehyde (4)

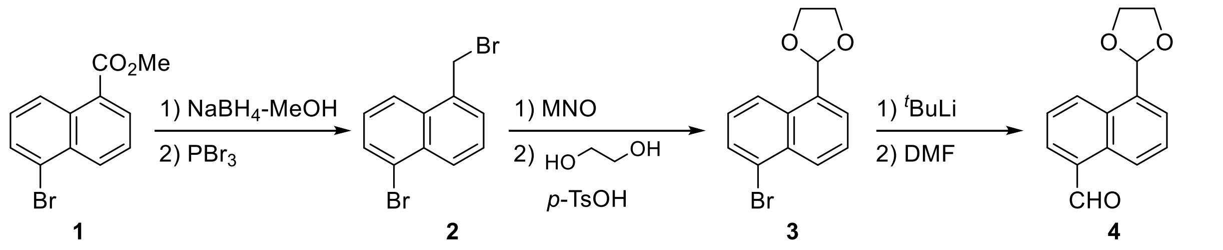

5-Bromo-1-naphthalenemethanol [S2,S3]

A suspension of methyl-5-bromo-1-naphthoate (5.0 g, 19 mmol) and $NaBH_4$ (1.8 g, 48 mmol, 2.5 equiv) in THF (75 mL) was heated to 63 °C under N_2, and methanol (3.5 mL, 95 mmol, 5 equiv) was slowly added. The mixture was heated at reflux overnight. The reaction mixture

was poured into ice water (100 mL), and extracted with AcOEt (2 × 80 mL). The organic layers were combined, washed with brine (80 mL) and dried (Na_2SO_4). The solvent was removed to afford compound 5-bromo-1-naphthalenemethanol as white solid (3.27 g, 73%).

M.p. 124–126°C (lit. 123–124°C)[S2]

^{1}H NMR (600 MHz, $CDCl_3$) δ_H = 8.25 (d, *J* = 8.2 Hz, 1H), 8.11 (d, *J* = 8.5 Hz, 1H), 7.82 (d, *J* = 7.4 Hz, 1H), 7.59–7.55 (m, 2H), 7.39 (t, *J* = 8.2 Hz, 1H), 5.15 (s, 2H), 1.65 (s, 1H).

^{13}C NMR (151 MHz, $CDCl_3$) δ_C = 136.8, 132.7, 132.5, 130.2, 127.9, 127.0, 126.7, 126.3, 123.8, 63.8.

1-Bromo-5-(bromomethyl)naphthalene (2) [S4]

5-Bromo-1-naphthalenemethanol (500 mg, 2.11 mmol) was dissolved in toluene (7 mL) and PBr_3 (175 µL, 1.84 mmol) was added at 0 °C. The mixture was heated at reflux overnight. After being cooled to rt, toluene (7 mL) was added. The resulting mixture was washed with water (15 mL) and the organic phase was collected. The aqueous layer was extracted with toluene (3 × 15 mL). The combined organic layers were dried (Na_2SO_4) and concentrated. The residue was purified by column chromatography (silica gel, toluene) to afford compound **2** as white solid (560 mg, 89%).

M.p. 101°C. (lit. 101°C) [S4]

^{1}H NMR (600MHz, $CDCl_3$) δ_H = 8.29 (d, *J* = 8.6 Hz, 1 H), 8.14 (d, *J* = 8.5 Hz, 1H), 7.84 (d, *J* = 7.4 Hz, 1H), 7.60 (d, *J* = 7.0 Hz, 1H), 7.52 (dd, *J* = 8.7, 7.1Hz, 1H), 7.46 (t, *J* = 7.4 Hz, 1H), 4.95 (s, 2H).

^{13}C NMR (151 MHz, $CDCl_3$) δ_C = 133.9, 132.7, 132.5, 130.6, 129.1, 128.7, 127.0, 126.9, 123.9, 123.8, 31.4.

5-Bromo-1-naphthaldehyde [S5]

1-Bromo-5-(bromomethyl)naphthalene (**2**, 500 mg, 1.67 mmol) was dissolved in THF (10 mL), and *N*-methylmorpholine *N*-oxide (NMO, 780 mg, 6.68 mmol) was added. The mixture was heated at reflux overnight under N_2. After cooling to rt, water (10 mL) was added. The mixture was extracted with toluene (3 × 20 mL). The organic layers were combined and washed with water (40 mL), dried (Na_2SO_4) and concentrated to give yellow solid (340 mg) which was purified by column chromatography (silica gel, toluene/AcOEt = 4/1) to afford 5-bromo-1-naphthaldehyde as white solid (320 mg, 82 %).

M.p. 106°C (lit. 104°C) [S5]

^{1}H NMR (600 MHz, $CDCl_3$) δ_H = 10.41 (s, 1H), 9.28 (d, *J* = 8.6 Hz, 1H), 8.59 (d, *J* = 8.5 Hz, 1H), 8.05 (d, *J* = 7.0 Hz, 1H), 7.90 (d, *J* = 7.4 Hz, 1H), 7.76 (t, *J* = 7.0 Hz, 1H), 7.53 (t, *J* = 7.5 Hz, 1H).

^{13}C NMR (151 MHz, $CDCl_3$) δ_C = 193.1, 137.4, 134.4, 132.4, 132.1, 131.7, 131.4, 129.5, 126.4, 124.9, 123.5.

2-(5-Bromonaphthalen-1-yl)-1,3-dioxolane (3)

5-Bromo-1-naphthaldehyde (800 mg, 3.41 mmol), ethylene glycol (474 μl, 8.55 mmol, 2.5 equiv) and *p*-TsOH·H_2O (1.44 mg, 7.57 μmol) were dissolved in toluene (35 mL) and heated at reflux for 48 h with a dean-stark trap. The resulting solution was washed with sat. $NaHCO_3$ aq. (50 mL) and the organic layer was collected. The aqueous layer was extracted with toluene (2 × 30 mL). The organic layers were combined, washed by brine (50 mL) and dried (Na_2SO_4). The solvent was removed to afford compound **3** as pale yellow solid (930 mg, 98%).

M.p. 77–79°C

^{1}H NMR (600 MHz, $CDCl_3$) δ_H = 8.31 (d, *J* = 8.6 Hz, 1H), 8.21 (d, *J* = 8.4 Hz, 1H), 7.83 (d, *J* = 7.1 Hz, 1H), 7.81 (dd, *J* = 7.3, 0.7 Hz, 1H), 7.59 (dd, *J* = 7.3, 1.4 Hz, 1H), 7.38 (dd, *J* = 7.5, 1.0 Hz, 1H), 6.46 (s, 1H), 4.23–4.13 (m, 4H).

^{13}**C NMR** (151 MHz, $CDCl_3$) δ_C = 133.7, 132.4, 132.4, 130.1, 128.8, 126.7, 126.6, 124.5, 124.1, 123.6, 102.0, 65.5.

HRMS (FAB+, $[M-H]^+$) Calcd. for $C_{13}H_{10}{}^{79}BrO_2$:276.9864. Found: 276.9863.

5-(1,3-Dioxolan-2-yl)-1-naphthaldehyde (4)

Compound **3** (1.04 g, 3.73 mmol) was put into a flame-dried flask. After vacuum drying for 10 min, the flask was filled with Ar. Dry THF (120 mL) was added into the flask and the resulting solution was cooled to −78 °C. Then, *t*-BuLi (1.6 M in pentane, 4.66 mL, 7.46 mmol, 2 equiv) was slowly added and the resulting mixture was stirred at the temperature for 1 h. Dry DMF (2 mL, 26 mmol) was added and the resulting mixture was stirred at −78 °C for 30 min. The mixture was then warmed to rt and stirred overnight. The mixture was diluted with water (80 mL) and extracted with CH_2Cl_2 (3 × 80 mL). The organic layers were combined, dried (Na_2SO_4) and concentrated to give a yellow oil. The oil was purified by column chromatography (silica gel, hexane/AcOEt = 9/1) followed by purification with preparative liquid chromatography (silica gel, hexane/AcOEt = 5/1) to afford compound **3** as yellowish solid (250 mg, 29%).

M.p. 81 °C

^{1}**H NMR** (600 MHz, $CDCl_3$) δ_H = 10.41 (s, 1H), 9.31 (d, *J* = 8.8 Hz, 1H), 8.54 (d, *J* = 8.8 Hz, 1H), 8.02 (d, *J* = 7.3 Hz, 1H), 7.88 (d, *J* = 7.8 Hz, 1H), 7.67–7.73 (m, 2H), 6.47 (s, 1H), 4.24–4.14 (m, 4H).

^{13}**C NMR** (151 MHz, $CDCl_3$) δ_C = 193.7, 136.4, 133.7, 131.8, 131.5, 131.4, 131.2, 128.4, 126.3, 125.4, 124.8, 102.3, 65.5.

Anal: Calcd for $C_{14}H_{12}O_3$: C, 73.67; H, 5.30. Found: C, 73.63; H, 5.37.

1-3 Synthesis of (2,3,4,5-tetrafluorobenzyl)triphenylphosphonium bromide (5) [S6]

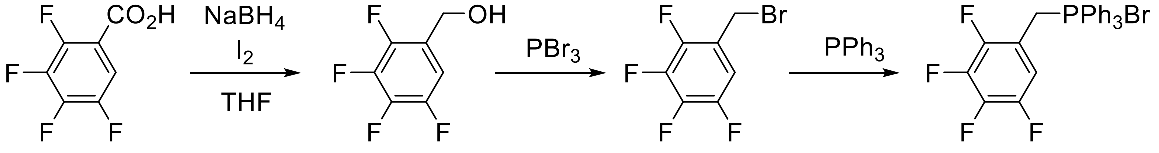

5

2,3,4,5-Tetrafluorobenzyl alcohol

To a suspension of $NaBH_4$ (0.27 g, 7.2 mmol) in THF (25 mL) was dropwise added a solution of 2,3,4,5-tetrafluorobenzoic acid (1.00 g, 5.15 mmol) in THF (10 mL). The mixture was stirred under an Ar atmosphere until gas evolution ceased. A solution of I_2 (0.70 g, 2.8 mmol, 0.55 equiv) in THF (10 mL) was dropwise added to the mixture. The mixture was stirred at 55 °C overnight. The reaction was quenched with MeOH (2.5 mL) and the solvent was removed. The residual dark green oil was triturated with $CHCl_3$ (25 mL) and the white solid formed was filtered off. The filtrate was washed with 1% aq. $Na_2S_2O_3$ (25 mL) and the organic layer was collected. The aqueous layer was extracted with $CHCl_3$ (25 mL). The combined organic phase and the extract were dried (Na_2SO_4) and concentrated to afford 2,3,4,5-tetrafluorobenzyl alcohol as colorless oil (0.59 g, 63%). The crude product was purified by distillation under reduced pressure.

B.p. 89°C/3.6 × 10^3 Pa (lit. 82–85°C/1 × 10^3 Pa) [S6].

^{1}H NMR (600 MHz, $CDCl_3$) δ_H = 7.15–7.05 (m, 1H), 4.75 (s, 1H).

^{13}C NMR (151 MHz, $CDCl_3$) δ_C = 110.0, 109.8, 58.1.

2,3,4,5-Tetrafluorobenzyl bromide

2,3,4,5-Tetrafluorobenzyl alcohol (160 mg, 0.884 mmol) was dissolved in Et_2O (4 mL) and PBr_3 (93 μL, 0.98 mmol, 1.1 equiv) was added at 0 °C. The mixture was stirred for 3 h at 0 °C and then stirred overnight at rt. The reaction was quenched with water (1 mL), and extracted

with Et_2O (3 × 2 mL). The organic layers were combined, dried (Na_2SO_4), and concentrated to afford **16** as colorless oil (150 mg, 68%).

^{1}H NMR (600 MHz, $CDCl_3$) δ_H = 7.07–7.02 (m, 1H), 4.43 (d, J = 1.4 Hz, 2H).

^{13}C NMR (151 MHz, $CDCl_3$) δ_C = 112.1, 112.0, 23.2.

^{19}F NMR (564 MHz, $CDCl_3$) δ_F = −139.5, −142.7, −155.3, −155.7.

(2,3,4,5-Tetrafluorobenzyl)triphenylphosphonium bromide (5)

A solution of 2,3,4,5-tetraflorobenzyl bromide (**16**, 750 mg, 3.09 mmol) and PPh_3 (890 mg, 3.39 mmol) in toluene (75 mL) was heated at reflux overnight. After concentration to one third of the volume, the white solid formed was collected by filtration to afford phosphonium salt **5** (1.44 g, 92%). The crude product was purified by recrystallization (CH_2Cl_2 and toluene).

M.p. 223–224°C (lit. 222.5–223.9°C) [S6]

^{1}H NMR (600 MHz, $CDCl_3$) δ_H = 7.87–7.78 (m, 9 H), 7.73–7.63 (m, 6 H), 7.44–7.33 (m, 1H), 5.68 (d, $J_{P\text{–}H}$ = 14.5 Hz, 2H).

^{13}C NMR (151 MHz, $CDCl_3$) δ_C = 135.6 (d, J_{CP} = 2.9 Hz), 134.3 (d, J_{CP} = 10.1Hz), 130.6 (d, J_{CP} = 12.7 Hz), 117.0 (d, J_{CP} = 86.5 Hz), 24.2 (d, J_{CP} = 48.6 Hz).

^{19}F NMR (564 MHz, $CDCl_3$) δ_F = −137.4, −140.4, −154.5, −155.9.

2.3. Synthesis of 5,6,7,8-tetrafluorophenanthrene-1-carbaldehyde (10)

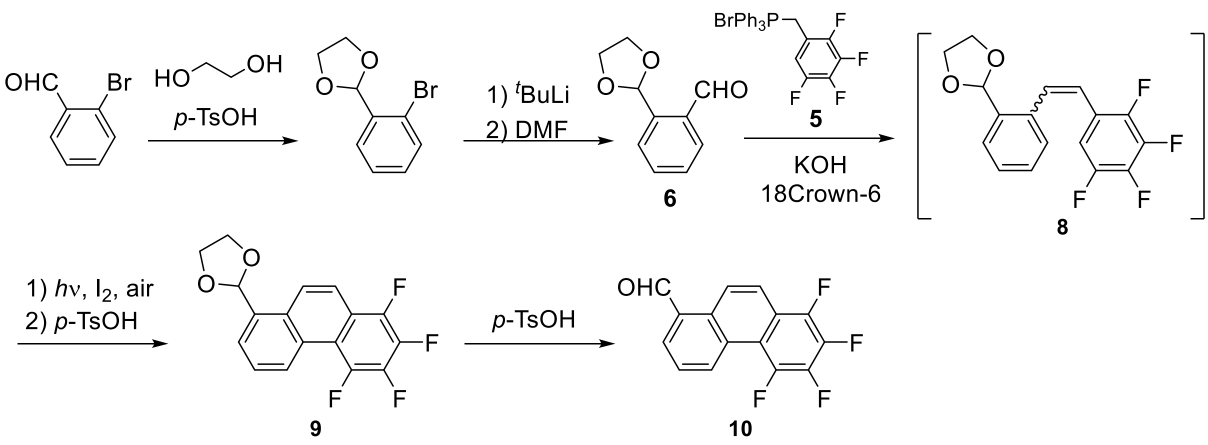

2-(2-Bromophenyl)-1,3-dioxolane [S7]

A solution of 2-bromobenzaldehyde (1.0 g, 5.4 mmol), ethylene glycol (0.84 g, 14 mmol, 2.5 equiv) and *p*-TsOH·H_2O (catalytic amount) in toluene (40 mL) was heated at reflux overnight using a dean-stark trap. The resulting mixture was washed with sat. aq. $NaHCO_3$ (50 mL) and the toluene layer was collected. The aqueous layer was extracted with toluene (2 × 40 mL). The toluene solutions were combined, dried (Na_2SO_4), and concentrated to afford 2-(2-bromophenyl)-1,3-dioxolane as colorless oil (1.24 g, quant.).

^{1}H NMR (600 MHz, $CDCl_3$) δ_H = 7.60 (d, *J* = 7.9 Hz, 1H), 7.56 (d, *J* = 7.9 Hz, 1H), 7.34 (t, *J* = 7.6 Hz, 1H), 7.22 (t, *J* = 7.6 Hz, 1H), 6.10 (s, 1H), 4.21–4.02 (m, 4 H).

^{13}C NMR (151 MHz, $CDCl_3$) δ_C = 136.8, 133.1, 130.8, 128.0, 127.6, 123.1, 102.8, 65.6.

2-(1,3-Dioxolan-2-yl)benzaldehyde (6) [S8]

2-(2-Bromophenyl)-1,3-dioxolane (1.24 g, 5.41 mmol) was put in a frame-dried flask, dried under reduced pressure for 10 min, and dry THF (150 mL) was added to the flask. Under an Ar atmosphere, *t*-BuLi (1.6 M in pentane, 7.5 mL, 12 mmol, 2.2 equiv) was dropwise added to the solution at −78 °C. The resulting mixture was stirred at −78 °C for 1 h, then DMF (5 mL) was added. The mixture was stirred for 30 min at the temperature. The resulting mixture was allowed to warm to rt and stirred overnight. The mixture was diluted with water (100 mL) and extracted with AcOEt (2 × 100 mL). The combined organic layers were concentrated to afford crude aldehyde as yellow oil. The crude oil was purified by column chromatography (silica gel, hexane/AcOEt = 9/1) to afford aldehyde **6** as colorless oil (70 mg, 7%).

^{1}H NMR (600 MHz, $CDCl_3$) δ_H = 10.41 (s, 1H), 7.94 (dd, *J* = 7.6, 1.4 Hz, 1H), 7.73 (d, *J* = 7.9 Hz, 1H), 7.62 (dt, *J* = 7.6, 1.4 Hz, 1H), 7.53 (t, J = 7.6 Hz), 6.41 (s, 1H), 4.20–4.06 (m, 4H).

^{13}C NMR (151 MHz, $CDCl_3$) δ_C = 191.8, 139.0, 134.4, 133.6, 130.1, 129.4, 126.9, 101.0, 65.3.

5,6,7,8-Tetrafluorophenanthrene-1-carbaldehyde (10)

(2,3,4,5-Tetrafluorobenzyl)triphenylphosphonium bromide (**5**, 1.42 g, 2.81 mmol), 2-(1,3-dioxolan-2-yl)benzaldehyde (**6**, 500 mg, 2.81 mmol), and 18-crown-6 (145 mg, 0.549 mmol) were dissolved in CH_2Cl_2 (50 mL), and KOH (200 mg, 3.56 mmol in H_2O) was added. The mixture was stirred at rt under Ar atmosphere for 5 h. The resulting mixture was washed with brine (20 mL) and the organic layer was collected, dried (Na_2SO_4), and concentrated. The residue was passed through a silica-gel pad (toluene) to afford compound **8** as colorless oil (*E/Z* mixture, *E*/*Z* ≈ 1:2, 617 mg, 68%) which was used for the next reaction without separation.

The *E*/*Z* mixture of compound **8** (50 mg, 0.15 mmol) was dissolved in cyclohexane (150 mL) and I_2 (small pieces) was added. The mixture was irradiated with black-light lamps (6 × 15 W, 306 nm) for 24 h. The solvent was removed and the residue was dissolved in toluene (50 mL). The toluene solution was washed with 1% aq. $Na_2S_2O_3$ (30 mL), dried (Na_2SO_4) and concentrated to afford a yellowish solid (50 mg). The solid was purified by column chromatography (silica gel, hexane/$CHCl_3$ = 7/3) to afford **9** as white solid (50 mg).

The obtained crude **9** was dissolved in acetone (5 mL) and *p*-TsOH·H_2O (30 mg, 0.16 mmol) was added. The mixture was heated at reflux for 4 h. To the resulting mixture was added water (5 mL) and extracted by toluene (2 × 10 mL). The combined organic layers were washed with brine (10 mL) and dried (Na_2SO_4). The solvent was removed to afford a yellow solid (60 mg) which was purified by column chromatography (silica gel, toluene) to afford aldehyde **10** as yellow solid (30 mg, 68%). An analytical sample was made by recrystallization ($CHCl_3$ and MeOH).

M.p. 167–168°C

^{1}H NMR (600 MHz, $CDCl_3$) δ_H = 10.47 (s, 1H), 9.31 (d, *J* = 9.3 Hz, 1H), 9.24–9.30 (br, 1H), 8.17 (d, *J* = 7.2 Hz, 1H), 8.12 (d, *J* = 9.3 Hz, 1H), 7.90 (t, *J* = 7.3 Hz, 1H).

^{13}C NMR (151 MHz, $CDCl_3$) δ_C = 193.3, 136.5, 133.3, 133.1, 131.6, 131.0, 127.4, 124.4, 120.3.

^{19}F NMR (564 MHz, $CDCl_3$) δ_F = −139.9, −149.2, −157.5, −157.8.

HRMS (FAB, $[M]^+$) Calcd. for $C_{15}H_6F_4O$: 278.0355. Found; 278.0337

1-4. Synthesis of 7,8,9,10-tetrafluorochrysene-1-carbaldehyde (13)

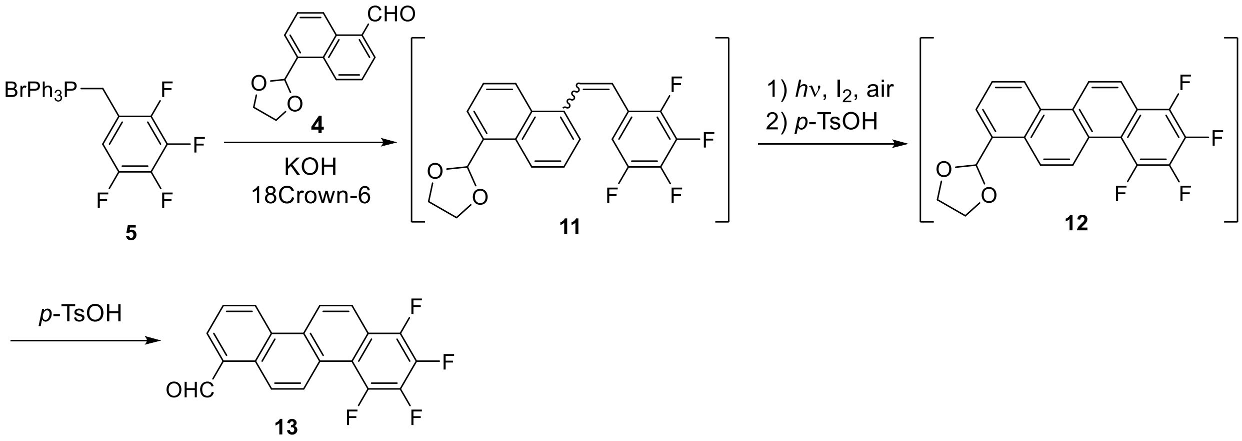

(2,3,4,5-Tetrafluorobenzyl)triphenylphosphonium bromide (**5**, 288 mg, 0.570 mmol), 5-(1,3-dioxolan-2-yl)-1-naphthaldehyde (**4**, 130 mg, 0.570 mmol), 18-crown-6 (17 mg, 64 μmmol), and KOH (40 mg, 0.71 mmol) were added to CH_2Cl_2 (20 mL) and heated at reflux under Ar overnight. The resulting mixture was washed with brine (20 mL) and the organic layer was collected. The aqueous layer was extracted with CH_2Cl_2 (20 mL). The combined organic layers were dried (Na_2SO_4) and concentrated. The residue was passed through a silica-gel pad (toluene) to obtain compound **11** as pale green solid as an *E*/*Z* mixture (*E*/*Z* ≈ 1:1.5) which was used for the next reaction without separation.

Compound **11** (200 mg, 0.534 mmol) and I_2 (162 mg, 0.638 mmol) were dissolved in a mixture of toluene (255 mL) and THF (45 mL). The solution was irradiated with black-light lamps (6 × 15 W, 352 nm) under N_2 overnight. The solution was concentrated to about 50 mL, washed with 1% aq. $Na_2S_2O_3$ (50 mL) and the organic phase was collected. The aqueous layer was extracted with toluene (50 mL). The combined organic layers were washed with brine (50 mL), dried (Na_2SO_4) and concentrated to obtain a pale yellow solid. The solid was recrystallized from a toluene–hexane mixture to afford compound **12** as pale yellow plates (150 mg, 81%). The product **12** was subjected to the following acid-promoted acetal deprotection without further purification.

The crude **12** (170 mg, 0.457 mmol), *p*-TsOH·H_2O (9 mg, 0.5 mmol) were dissolved in acetone (70 mL) and heated at reflux overnight. The mixture was washed with water (70 mL) and the organic phase was collected. The aqueous layer was extracted with toluene (3 × 70 mL). The combined organic layers were washed with brine (50 mL), dried (Na_2SO_4) and concentrated. The residual oil was purified by chromatography (silica gel column, toluene) to afford compound **13** as yellow solid (110 mg, 73%). An analytical sample was prepared by recrystallization from $CHCl_3$ as white solid.

M.p. 227–228°C

^{1}H NMR (600 MHz, $CDCl_3$) δ_H = 10.58 (s, 1H), 9.45 (d, J = 9.6 Hz, 1H), 9.21 (dd, J = 9.6, 2.4 Hz, 1H), 9.08 (d, J = 8.4 Hz, 1H), 8.83 (d, J = 9.0 Hz, 1H), 8.25 (dd, J = 9.6 Hz, 1.2 Hz, 1H), 8.21 (d, J = 7.2 Hz, 1H), 7.92 (dd, J = 8.4, 7.2 Hz, 1H).

^{13}C NMR (151 MHz, $CDCl_3$) δ_C = 193.1, 136.0, 132.0, 130.7, 130.4, 129.7, 129.3, 127.6, 127.4, 126.4, 125.0, 124.7, 123.2, 118.7, 116.3.

HRMS (FAB+, $[M-H]^+$) Calcd. for $C_{15}H_6F_4O$:278.0355. Found: 278.0337.

1-5. Synthesi of 1-bromo-5,6,7,8-tetrafluorophenanthrene (15)

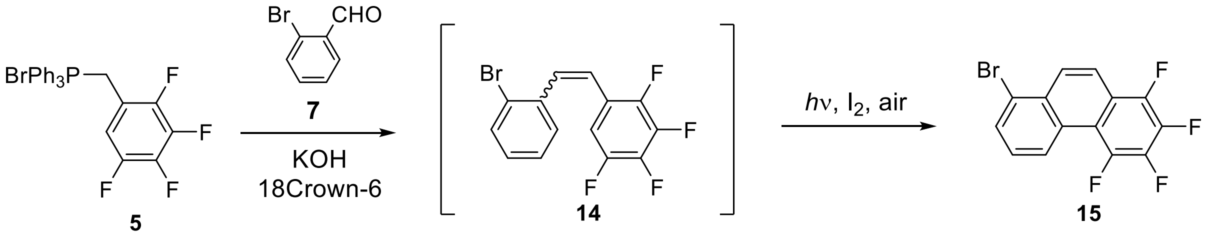

A mixture of phosphonium salt **5** (227 mg, 0.449 mmol), 2-bromobenzaldehyde (**7**, 83.2 mg, 0.450 mmol), 18-crown-6 (22 mg, 83 μmol) and KOH (100 mg, 1.78 mmol) in CH_2Cl_2 (30 mL) was heated at reflux under Ar for 72 h. An additional portion of phosphonium bromide **5** (120 mg, 0.237 mmol), 18-crown-6 (30 mg, 0.11 mmol) in CH_2Cl_2 (14 mL) was added and further heated at reflux overnight. The mixture was washed with brine (30 mL) and the organic phase was collected. The aqueous layer was extracted with CH_2Cl_2 (30 mL). The combined

organic phases were concentrated. The residue was passed through a silica-gel pad (hexane/$CHCl_3$ = 4/1) to afford compound **14** as a white solid as an *E/Z* mixture (*E/Z* ≈ 1:7), which was used for the next reaction without separation.

The crude **14** (180 mg, 0.544 mmol) and I_2 (small pieces) were dissolved in cyclohexane (500 mL) and the solution was irradiated with black-light lamps (6 × 15 W, 306 nm) overnight. The solvent was removed and the residue was dissolved in toluene (100 mL). The solution was washed with 1% aq. $Na_2S_2O_3$ (50 mL) and the organic phase was collected. The aqueous layer was extracted with toluene (50 mL). The combined organic layers were washed with brine (20 mL), dried (Na_2SO_4) and concentrated to afford compound **15** as white solid (180 mg, quant.).

M.p. 152–153°C

^{1}H NMR (600 MHz, $CDCl_3$) δ_H = 8.99 (dd, *J* = 8.6, 3.1 Hz, 1H), 8.34 (d, *J* = 9.3 Hz, 1H), 8.02 (d, *J* = 9.3 Hz, 1H), 7.98 (d, *J* = 7.6 Hz, 1H), 7.56 (t, *J* = 7.6 Hz, 1H).

^{13}C NMR (151 MHz, $CDCl_3$) δ_C = 132.4, 131.0, 129.0, 128.5, 127.7, 126.8, 126.7, 124.0, 118.7, 115.7.

Anal. Calcd. for $C_{14}H_5BrF_4$: C, 51.10; H, 1.53. Found: C, 50.98; H, 1.55.

1-6. Synthesis of octafluorinated phenacenens

1-6-1. 1,2,3,4,9,10,11,12-Octafluoropicene (F_8PIC)

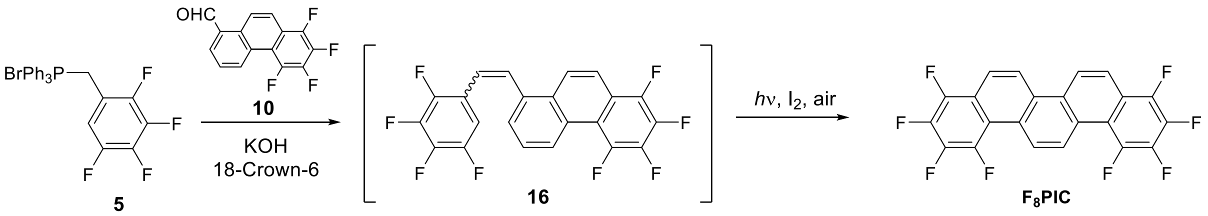

Tetrafluorophenanthrene-1-carbaldehyde **10** (70 mg, 0.25 mmol), phosphonium bromide **5** (141 mg, 0.279 mmol), 18-crown-6 (12 mg, 45 μmol) and KOH (100 mg, 1.78 mmol) were added in CH_2Cl_2 (50 mL). The mixture was heated at reflux under Ar for 120 h. An additional

amount of phosphonium bromide **5** (71 mg, 0.14 mmol) and KOH (100 mg) were added and the mixture was further heated at reflux for 96 h. The resulting mixture was washed with brine (20 mL) and the aqueous layer was extracted with CH_2Cl_2 (20 mL). The combined organic layers were concentrated to give a yellow solid which was passed through a silica-gel pad (toluene) to afford compound **16** as an *E/Z* mixture (*E/Z* ≈ 1:1), which was used for the next reaction without separation.

The crude compound **16** (105 mg, 0.247 mmol) and I_2 (small pieces) were dissolved in toluene (250 mL) and irradiated with black-light lamps (6 × 15 W, 352 nm) overnight. The mixture was concentrated to ca. 150 mL and washed with 1% aq. $Na_2S_2O_3$ (50 mL). The organic layer was collected, dried (Na_2SO_4) and concentrated to obtain the crude product which was washed with $CHCl_3$ to afford **F$_8$PIC** as pale yellow solid (64 mg, 61%). An analytical sample was prepared by sublimation under reduced pressure (107 Pa, 165 °C).

M.p. 284°C

^{1}H NMR (600 MHz, $CDCl_3$, 50°C) δ_H = 9.28 (d, *J* = 8.7 Hz, 2H), 8.86 (s, 2H), 8.25 (d, *J* = 8.9 Hz, 1H).

^{13}C NMR (151 MHz, $CDCl_3$, 50°C) δ_C = 128.3, 126.6, 126.5, 123.5, 120.3, 118.6.

^{19}F NMR (564 MHz, $CDCl_3$, 50°C) δ_F = −139.7, −149.7, −157.7, −157.9.

Anal. Calcd. for $C_{22}H_6F_8$: C, 62.58; H, 1.43. Found: C, 62.31; H, 1.43.

1-6-2. 1,2,3,4,9,10,11,12-Octafluorofulminene (F$_8$FUL)

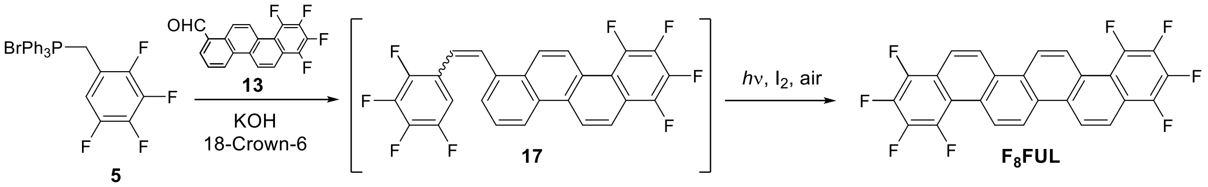

A mixture of phosphonium bromide **5** (167 mg, 0.331 mmol), chrysene-1-carbaldehyde **13** (100 mg, 0.304 mmol), 18-crown-6 (15 mg, 57 mmol) and KOH (60 mg, 1.1 mmol) in CH_2Cl_2

(50 mL) was heated at reflux under Ar for 72 h. The solvent was removed and the residue was treated with toluene (50 mL). The precipitate formed was collected by filtration and washed with $CHCl_3$ (A: white solid, 97 mg). The filtrate was washed with brine (30 mL) and the organic layer was collected. The aqueous layer was extracted with toluene (30 mL). The combined organic layers were dried (Na_2SO_4) and concentrated. The residual solid was passed through a silica-gel pad (toluene) to afford an additional portion of the title compound as yellow solid (B: 30 mg). The total yield of **17** (A + B) was 127 mg as an *E/Z* mixture (*E/Z* ≈ 1:4). The crude mixture was used for the next reaction without separation.

Compound **17** (127 mg, 0.268 mmol), I_2 (small piece) were dissolved in toluene (700 mL), and the solution was irradiated with black-light lamps (6 × 15 W, 352 nm) at refluxing temperature overnight. The mixture was cooled and concentrated to about 100 mL, and the precipitate formed was collected by filtration (pale yellow solid: A). The filtrate was washed with 1% aq. $Na_2S_2O_3$ (50 mL) and the organic phase was collected. The aqueous layer was extracted with toluene (50 mL). The combined organic layers were dried (Na_2SO_4) and concentrated. The residue was washed with small amounts of toluene and EtOH to obtain a pale yellow solid (B). Total yield of **F$_8$FUL**: (A) + (B) was 74 mg (52%). An analytical sample was obtained by sublimation under reduced pressure (100 Pa, 250°C) as pale yellow solid.

M.p. >300°C

^{1}H NMR (600 MHz, tetrachloroethane-*d*$_2$, 120°C) δ_H = 9.35 (d, *J* = 9.0 Hz, 2H), 9.08 (d, *J* = 9.6 Hz, 2H), 9.02 (d, *J* = 9 Hz, 2H), 8.34 (d, *J* = 8.4 Hz, 2H).

^{19}F NMR (564 MHz, tetrachloroethane-*d*$_2$, 120°C) δ_F = −140.1, −149.5, −158.0, −158.4.

^{13}C NMR spectrum was not obtained due to low solubility.

Anal. Calcd. for $C_{24}H_8F_4$: C, 66.12; H, 1.71. Found: C, 66.16; H, 1.68.

1-6-3. 1,2,3,4,11,12,13,14-Octafluoro[7]phenacene ($F_8$7PHEN)

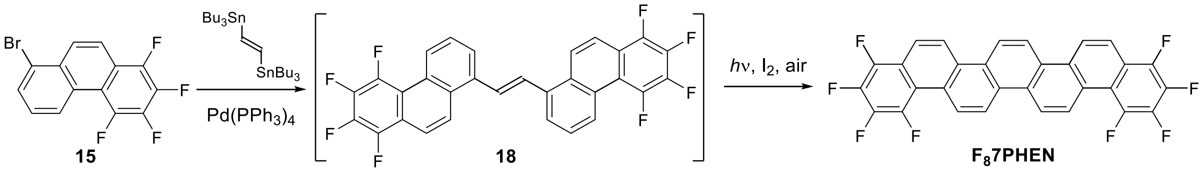

A solution of tetrafluorophenanthrene **15**(165 mg, 0.498 mmol), $Pd(PPh_3)_4$ (29 mg, 25 μmol) and (*E*)-1,2-bis(tributylstannyl)ethylene (151 mg, 0.249 mmol) in dry toluene (15 mL) was heated at reflux overnight under Ar. The black precipitate formed was collected by filtration. The obtained solid was dissolved in toluene (500 mL) and I_2 (small pieces) was added. The solution was irradiated with black-light lamps (6 × 15 W, 352 nm) at 150 °C for 48 h. The precipitate formed was collected by filtration and purified by sublimation under reduced pressure (99 Pa, 300 °C) to afford **F87PHN** as yellow solid (52 mg, 20%).

M.p. ＞300°C

^{1}H NMR (600 MHz, tetrachloroethane-*d*$_2$, 100°C) δ_H = 9.37 (d, *J* = 9.2 Hz, 2H), 9.18 (d, *J* = 9.1 Hz, 2H), 9.08 (s, 2H), 9.03 (d, *J* = 9.2 Hz, 2H), 8.34 (d, *J* = 9.2 Hz, 2H).

^{13}C NMR spectrum was not obtained due to low solubility.

Anal. Calcd for $C_{26}H_{10}F_8$: C, 68.98; H, 1.93. Found: C, 69.26; H, 2.49.

1-7. Synthesis of parent phenacenes

The parent picene (**PIC**) [S9], fulminene (**FUL**) [S10], and [7]phenacene (**7PHEN**) [S11] were prepared according to the previously reported procedures.

2. Fluorescence excitation spectra of F8PHENs.

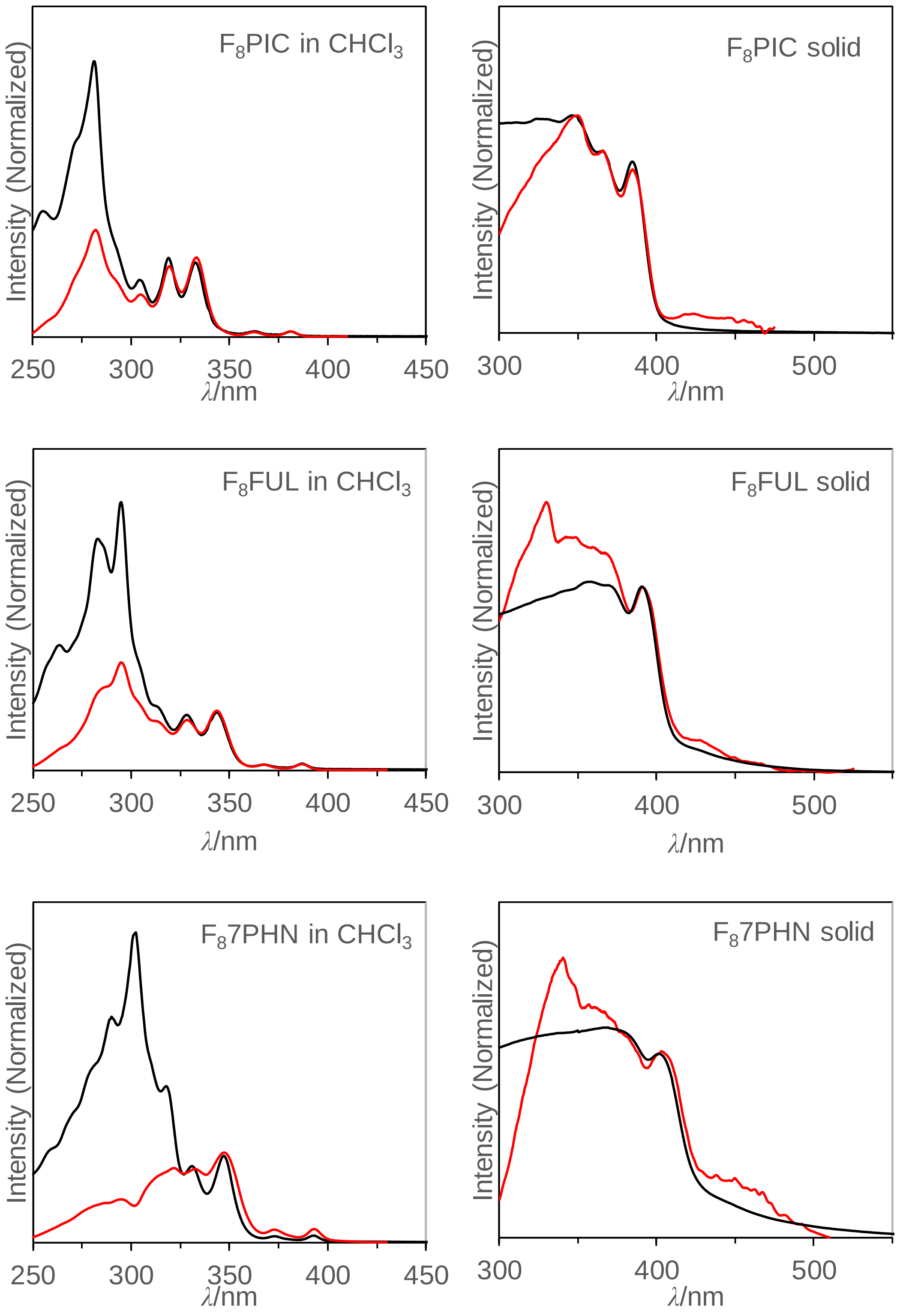

Figure S1. Absorption (—) and excitation (—) spectra of **F8PIC**, **F8FUL** and **F87PHN** in $CHCl_3$ and in solid state.

3. Theoretical calculations

The theoretical calculations were performed by using Gaussian 16 [S12] package by using B3LYP functional [S13] and 6-31+G(d,p) basis set. The S_0 state geometries of the parent and fluorinated phenacenes were optimized in vacuum. The obtained Cartesian coordinates are summarized in Table S1(a)–(f). The vertical electronic transitions were calculated by the time-dependent (TD) method [S14] at the TD-B3LYP/6-31+G(d,p) level of theory. The calculated transitions ($S_0 \rightarrow S_n$, n =1–25) are summarized in Table S2(a)–(f). The electrostatic potentials in the S_0 state were mapped on the total electron density S_0 surfaces and illustrated in Figure 5 in the main article.

Table S1. Optimized geometries (in Cartesians/Å)

(a) PIC (E_e = -846.874268 au)

Atom	x	y	z
C	0.00000000	5.72988000	-0.38322500
C	0.00000000	5.00768300	0.79368600
C	0.00000000	3.59073100	0.77997800
C	0.00000000	2.89129400	-0.46371100
C	0.00000000	3.66324200	-1.65381700
C	0.00000000	5.04630900	-1.61704600
C	0.00000000	2.84078400	1.99691100
C	0.00000000	1.43595100	-0.45992300
C	0.00000000	0.72444800	0.76963400
C	0.00000000	1.47636800	1.99205900
C	0.00000000	-0.72444800	0.76963400
C	0.00000000	-1.43595100	-0.45992300
C	0.00000000	-0.68514900	-1.66865000
C	0.00000000	0.68514900	-1.66865000
H	0.00000000	3.38111400	2.94003900
H	0.00000000	6.81564200	-0.36169700
H	0.00000000	5.51894700	1.75304700
H	0.00000000	3.17366100	-2.62060500
H	0.00000000	5.60822800	-2.54654400
H	0.00000000	0.95995900	2.94351400
H	0.00000000	-1.19753400	-2.62313100
H	0.00000000	1.19753400	-2.62313100
C	0.00000000	-1.47636800	1.99205900
C	0.00000000	-2.84078400	1.99691100
H	0.00000000	-3.38111400	2.94003900
C	0.00000000	-3.59073100	0.77997800
C	0.00000000	-2.89129400	-0.46371100
H	0.00000000	-0.95995900	2.94351400
C	0.00000000	-5.00768300	0.79368600
C	0.00000000	-5.72988000	-0.38322500
H	0.00000000	-6.81564200	-0.36169700
C	0.00000000	-5.04630900	-1.61704600
H	0.00000000	-5.60822800	-2.54654400
C	0.00000000	-3.66324200	-1.65381700
H	0.00000000	-3.17366100	-2.62060500
H	0.00000000	-5.51894700	1.75304700

(b) FUL (E_e = -1000.52393 au)

Atom	x	y	z
C	1.76981400	0.71976800	-0.00000200
C	2.55030900	-0.46498700	-0.00001000
C	4.00264900	-0.38459300	-0.00001400
C	4.62851800	0.89807600	-0.00001100
C	3.80922200	2.06904700	-0.00000500
C	2.44703900	1.98413800	0.00000000
C	4.84258200	-1.52797800	-0.00002100
C	6.04246800	0.99399700	-0.00001500
C	6.83162800	-0.13894400	-0.00002200
C	6.22096600	-1.41065600	-0.00002400
H	4.29353000	3.04211600	-0.00000300
H	1.87455700	2.90310100	0.00000400
C	0.32210700	0.63398900	0.00000300

C	-0.50217100	1.79777200	0.00001300
C	-0.32210700	-0.63398900	-0.00000200
C	-1.86882500	1.71608100	0.00001800
H	-0.05208000	2.78251100	0.00001900
C	-1.76981400	-0.71976800	0.00000100
C	-2.55030900	0.46498700	0.00001100
H	-2.43603400	2.63904900	0.00002700
C	0.50217100	-1.79777200	-0.00001000
C	1.86882500	-1.71608100	-0.00001300
H	0.05208000	-2.78251100	-0.00001200
H	2.43603400	-2.63904900	-0.00001800
C	-4.00264900	0.38459300	0.00001500
C	-4.62851800	-0.89807600	0.00000800
C	-4.84258200	1.52797800	0.00002600
C	-6.04246800	-0.99399700	0.00001100
C	-6.22096600	1.41065600	0.00003000
C	-6.83162800	0.13894400	0.00002200
C	-3.80922200	-2.06904700	-0.00000400
C	-2.44703900	-1.98413800	-0.00000700
H	-4.29353000	-3.04211600	-0.00001000
H	-1.87455700	-2.90310100	-0.00001600
H	6.49720000	1.98139900	-0.00001300
H	7.91431100	-0.05447200	-0.00002500
H	6.83614200	-2.30579700	-0.00002900
H	4.40995800	-2.52156400	-0.00002300
H	-4.40995800	2.52156400	0.00003300
H	-6.83614200	2.30579700	0.00003900
H	-7.91431100	0.05447200	0.00002500
H	-6.49720000	-1.98139900	0.00000500

(c) 7PHEN (E_e = -1154.1765430 au)

Atom	x	y	z
C	-2.89091100	0.72850500	-0.00000300
C	-3.60121800	-0.50015000	0.00000600
C	-5.05596400	-0.50413100	0.00000700
C	-5.75511000	0.74003000	-0.00000100
C	-5.00526800	1.95684800	-0.00001000
C	-3.64053700	1.95149400	-0.00001100
C	-5.82805500	-1.69431300	0.00001600
C	-7.17222600	0.75380400	0.00000000
C	-7.89436600	-0.42304300	0.00000800
C	-7.21097600	-1.65715400	0.00001600
H	-5.54549100	2.90003500	-0.00001600
H	-3.12237200	2.90218100	-0.00001800
C	-1.44146400	0.72657800	-0.00000400
C	-0.68398800	1.93684500	-0.00001300
C	-0.72578600	-0.50050700	0.00000400
C	0.68399100	1.93684400	-0.00001400
H	-1.19078000	2.89368200	-0.00001900
C	0.72578600	-0.50050800	0.00000300
C	1.44146500	0.72657600	-0.00000600
H	1.19078500	2.89368000	-0.00002100
C	-1.47874600	-1.71058100	0.00001300
C	-2.84832400	-1.70912700	0.00001400
H	-0.97061600	-2.66683800	0.00002000
H	-3.36066400	-2.66367000	0.00002100
C	2.89091100	0.72850200	-0.00000700
C	3.60121800	-0.50015200	0.00000100
C	3.64053500	1.95149200	-0.00001600
C	5.05596400	-0.50413300	0.00000000
C	5.00526600	1.95684800	-0.00001700
C	5.75510900	0.74003000	-0.00000900
C	2.84832300	-1.70913000	0.00001000
C	1.47874400	-1.71058200	0.00001100
H	3.36066200	-2.66367200	0.00001600
H	0.97061500	-2.66683900	0.00001800
H	-7.68344500	1.71318500	-0.00000700
H	-8.98013800	-0.40154200	0.00000900
H	-7.77321300	-2.58647400	0.00002300
H	-5.33845800	-2.66111400	0.00002200
H	3.12236700	2.90217800	-0.00002200
H	5.54548800	2.90003500	-0.00002400
C	7.17222500	0.75380600	-0.00001000
C	7.89436600	-0.42304100	-0.00000300
C	5.82805700	-1.69431300	0.00000800
C	7.21097900	-1.65715200	0.00000600
H	7.68344300	1.71318700	-0.00001700
H	8.98013800	-0.40153700	-0.00000400

H	7.77321700	-2.58647200	0.00001200
H	5.33846200	-2.66111500	0.00001500

(d) F_8PIC (E_e = -1640.736165 au)

Atom	x	y	z
C	0.00000000	5.75680500	-0.02274600
C	0.00000000	4.98793500	1.11811900
C	0.00000000	3.57852800	1.06791200
C	0.00000000	2.90122400	-0.19446700
C	0.00000000	3.73040300	-1.34442900
C	0.00000000	5.11059700	-1.26711400
C	0.00000000	2.82208600	2.27795400
C	0.00000000	1.44252300	-0.20999200
C	0.00000000	0.72425500	1.01923200
C	0.00000000	1.46070300	2.24922700
C	0.00000000	-0.72425500	1.01923200
C	0.00000000	-1.44252300	-0.20999200
C	0.00000000	-0.68593500	-1.41505100
C	0.00000000	0.68593500	-1.41505100
H	0.00000000	3.35308700	3.22246300
H	0.00000000	0.93793800	3.19583700
H	0.00000000	-1.18858000	-2.36885600
H	0.00000000	1.18858000	-2.36885600
C	0.00000000	-1.46070300	2.24922700
C	0.00000000	-2.82208600	2.27795400
H	0.00000000	-3.35308700	3.22246300
C	0.00000000	-3.57852800	1.06791200
C	0.00000000	-2.90122400	-0.19446700
H	0.00000000	-0.93793800	3.19583700
C	0.00000000	-4.98793500	1.11811900
C	0.00000000	-5.75680500	-0.02274600
C	0.00000000	-5.11059700	-1.26711400
C	0.00000000	-3.73040300	-1.34442900
F	0.00000000	3.22447100	-2.59801900
F	0.00000000	5.84882800	-2.38602600
F	0.00000000	7.09489700	0.03183600
F	0.00000000	5.60847400	2.31650600
F	0.00000000	-3.22447100	-2.59801900
F	0.00000000	-5.84882800	-2.38602600
F	0.00000000	-7.09489700	0.03183600
F	0.00000000	-5.60847400	2.31650600

(e) F_8FUL (E_e = -1794.387740 au)

Atom	x	y	z
C	-1.74502000	0.77881600	0.00000200
C	-2.57126500	-0.37840500	0.00001100
C	-4.02094100	-0.22722700	0.00001500
C	-4.57799700	1.09359900	0.00001100
C	-3.71359000	2.22819100	0.00000300
C	-2.36015300	2.07165500	-0.00000100
C	-4.95476400	-1.29475700	0.00002200
C	-5.97729600	1.27424800	0.00001500
C	-6.84868700	0.21033800	0.00002200
C	-6.32104300	-1.08914000	0.00002600
H	-4.15476100	3.21785500	0.00000100
H	-1.74631000	2.96238200	-0.00000600
C	-0.30102100	0.64335100	-0.00000200
C	0.56403000	1.77442500	-0.00001200
C	0.30102100	-0.64335100	0.00000300
C	1.92803400	1.65307900	-0.00001600
H	0.15460600	2.77618400	-0.00001700
C	1.74502000	-0.77881600	-0.00000100
C	2.57126500	0.37840500	-0.00001100
H	2.51898900	2.55482900	-0.00002400
C	-0.56403000	-1.77442500	0.00001100
C	-1.92803400	-1.65307900	0.00001500
H	-0.15460600	-2.77618400	0.00001500
H	-2.51898900	-2.55482900	0.00002100
C	4.02094100	0.22722700	-0.00001500
C	4.57799700	-1.09359900	-0.00000800
C	4.95476400	1.29475700	-0.00002500
C	5.97729600	-1.27424800	-0.00001200
C	6.32104300	1.08914000	-0.00002800
C	6.84868700	-0.21033800	-0.00002200
C	3.71359000	-2.22819100	0.00000200
C	2.36015300	-2.07165500	0.00000500
H	4.15476100	-3.21785500	0.00000700
H	1.74631000	-2.96238200	0.00001300

F	4.56770500	2.59077500	-0.00003200
F	7.16107500	2.13450700	-0.00003800
F	8.17647600	-0.38868300	-0.00002500
F	6.48361100	-2.52529600	-0.00000600
F	-6.48361100	2.52529600	0.00001100
F	-8.17647600	0.38868300	0.00002600
F	-7.16107500	-2.13450700	0.00003300
F	-4.56770500	-2.59077500	0.00002600

(f) $F_8$7PHEN (E_e = -1948.039064 au)

Atom	x	y	z
C	0.00000000	2.89078100	-0.93797500
C	0.00000000	3.60650900	0.29220300
C	0.00000000	5.06443600	0.27552800
C	0.00000000	5.74175800	-0.98786800
C	0.00000000	4.98628700	-2.19811000
C	0.00000000	3.62439100	-2.16787800
C	0.00000000	5.89545200	1.42472600
C	0.00000000	7.15160800	-1.03793000
C	0.00000000	7.92100400	0.10208500
C	0.00000000	7.27515200	1.34687600
H	0.00000000	5.51759500	-3.14245500
H	0.00000000	3.09603900	-3.11193200
C	0.00000000	1.44196200	-0.93748500
C	0.00000000	0.68361700	-2.14836100
C	0.00000000	0.72596800	0.28784700
C	0.00000000	-0.68361700	-2.14836100
H	0.00000000	1.18622800	-3.10687500
C	0.00000000	-0.72596800	0.28784700
C	0.00000000	-1.44196200	-0.93748500
H	0.00000000	-1.18622800	-3.10687500
C	0.00000000	1.47856500	1.49426000
C	0.00000000	2.84888600	1.50140400
H	0.00000000	0.97240800	2.45129800
H	0.00000000	3.35347000	2.45419000
C	0.00000000	-2.89078100	-0.93797500
C	0.00000000	-3.60650900	0.29220300
C	0.00000000	-3.62439100	-2.16787800
C	0.00000000	-5.06443600	0.27552800
C	0.00000000	-4.98628700	-2.19811000
C	0.00000000	-5.74175800	-0.98786800
C	0.00000000	-2.84888600	1.50140400
C	0.00000000	-1.47856500	1.49426000
H	0.00000000	-3.35347000	2.45419000
H	0.00000000	-0.97240800	2.45129800
H	0.00000000	-3.09603900	-3.11193200
H	0.00000000	-5.51759500	-3.14245500
C	0.00000000	-7.15160800	-1.03793000
C	0.00000000	-7.92100400	0.10208500
C	0.00000000	-5.89545200	1.42472600
C	0.00000000	-7.27515200	1.34687600
F	0.00000000	7.77195700	-2.23686800
F	0.00000000	9.25967200	0.04717700
F	0.00000000	8.01440600	2.46579300
F	0.00000000	5.39029600	2.67943400
F	0.00000000	-5.39029600	2.67943400
F	0.00000000	-8.01440600	2.46579300
F	0.00000000	-9.25967200	0.04717700
F	0.00000000	-7.77195700	-2.23686800

Table S2. Vertical electronic transitions (S_0-S_n, n = 1–25) including the configurations for the S_0–S_1 and S_0–S_2 transitions.

(a) PIC

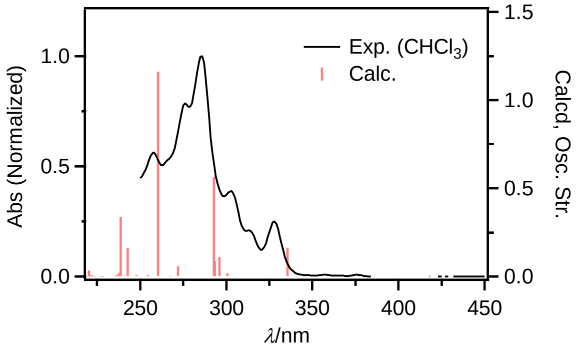

S_0–S_1 349.76 nm f = 0.0053
H−1 → L -0.40812
H → L+1 0.56164
S_0–S_2 335.50 nm f = 0.1602
H−1 → L+1 0.26524
H → L 0.64431
S_0–S_3 300.59 nm f = 0.0195
S_0–S_4 296.10 nm f = 0.1104
S_0–S_5 293.34 nm f = 0.0884
S_0–S_6 292.81 nm f = 0.5643
S_0–S_7 271.92 nm f = 0.0593
S_0–S_8 267.37 nm f = 0.0031
S_0–S_9 260.40 nm f = 1.1604
S_0–S_{10} 254.38 nm f = 0.0079
S_0–S_{11} 247.90 nm f = 0.0092
S_0–S_{12} 242.60 nm f = 0.1610
S_0–S_{13} 240.60 nm f = 0.0043
S_0–S_{14} 238.61 nm f = 0.3385
S_0–S_{15} 237.83 nm f = 0.0175
S_0–S_{16} 236.38 nm f = 0.0097
S_0–S_{17} 235.68 nm f = 0.0036
S_0–S_{18} 228.13 nm f = 0.0000
S_0–S_{19} 227.83 nm f = 0.0002
S_0–S_{20} 223.64 nm f = 0.0000
S_0–S_{21} 222.01 nm f = 0.0069
S_0–S_{22} 220.80 nm f = 0.0000
S_0–S_{23} 220.39 nm f = 0.0332
S_0–S_{24} 220.29 nm f = 0.0057
S_0–S_{25} 218.33 nm f = 0.0078

(b) FUL

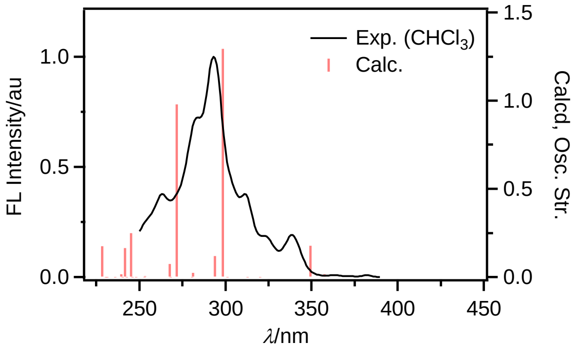

S_0–S_1 357.45 nm f = 0.0162
H−1 → L -0.37950
H−1 → L+1 0.12504
H → L 0.32560
H → L+1 0.47355
S_0–S_2 349.37 nm f = 0.1768
H−1 → L 0.17157
H−1 → L+1 0.18629
H → L 0.58001
H → L+1 -0.30713
S_0–S_3 320.22 nm f = 0.0000
S_0–S_4 312.76 nm f = 0.0000
S_0–S_5 301.16 nm f = 0.0000
S_0–S_6 298.57 nm f = 1.2947
S_0–S_7 293.86 nm f = 0.1192
S_0–S_8 281.20 nm f = 0.0238
S_0–S_9 280.54 nm f = 0.0000
S_0–S_{10} 271.56 nm f = 0.9779
S_0–S_{11} 268.22 nm f = 0.0000
S_0–S_{12} 267.70 nm f = 0.0753
S_0–S_{13} 253.32 nm f = 0.0054
S_0–S_{14} 253.07 nm f = 0.0000
S_0–S_{15} 248.05 nm f = 0.0000
S_0–S_{16} 246.00 nm f = 0.0000
S_0–S_{17} 245.15 nm f = 0.2476
S_0–S_{18} 242.80 nm f = 0.0000
S_0–S_{19} 241.68 nm f = 0.1653
S_0–S_{20} 240.23 nm f = 0.0000
S_0–S_{21} 239.57 nm f = 0.0153
S_0–S_{22} 235.97 nm f = 0.0000
S_0–S_{23} 231.36 nm f = 0.0000
S_0–S_{24} 230.61 nm f = 0.0000
S_0–S_{25} 228.28 nm f = 0.1738

(c) 7PHEN

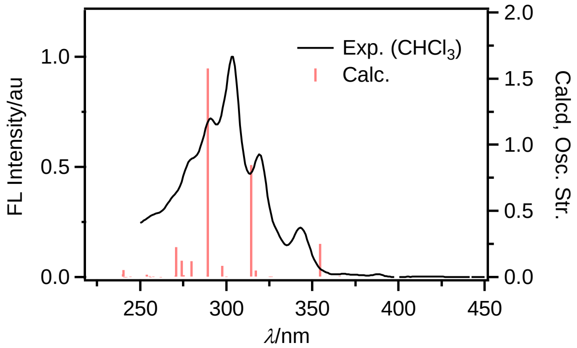

S_0–S_1	365.80 nm	f = 0.0085
	H−2 → L	0.13348
	H−1 → L	0.36859
	H → L+1	0.56388
	H → L+2	0.11231
S_0–S_2	354.54 nm	f = 0.2497
	H−1 → L+1	-0.23609
	H → L	0.65445
S_0–S_3	326.29 nm	f = 0.0050
S_0–S_4	325.29 nm	f = 0.0046
S_0–S_5	317.06 nm	f = 0.0492
S_0–S_6	314.55 nm	f = 0.8457
S_0–S_7	299.99 nm	f = 0.0047
S_0–S_8	297.59 nm	f = 0.0856
S_0–S_9	289.31 nm	f = 1.5757
S_0–S_{10}	289.29 nm	f = 0.0130
S_0–S_{11}	279.77 nm	f = 0.1199
S_0–S_{12}	275.17 nm	f = 0.0126
S_0–S_{13}	273.99 nm	f = 0.1235
S_0–S_{14}	270.91 nm	f = 0.2255
S_0–S_{15}	270.53 nm	f = 0.0120
S_0–S_{16}	262.04 nm	f = 0.0011
S_0–S_{17}	257.50 nm	f = 0.0027
S_0–S_{18}	255.98 nm	f = 0.0013
S_0–S_{19}	255.37 nm	f = 0.0085
S_0–S_{20}	253.90 nm	f = 0.0166
S_0–S_{21}	244.26 nm	f = 0.0018
S_0–S_{22}	241.81 nm	f = 0.0004
S_0–S_{23}	240.72 nm	f = 0.0001
S_0–S_{24}	240.31 nm	f = 0.0542
S_0–S_{25}	240.15 nm	f = 0.0200

(d) F_8PIC

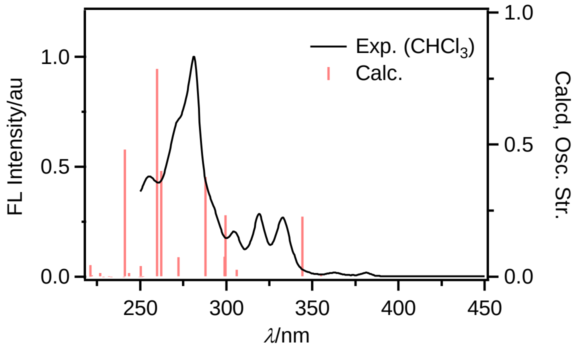

S_0–S_1	355.00 nm	f = 0.0109
	H−2 → L	0.12194
	H−1 → L	0.37615
	H → L+1	0.57841
S_0–S_2	344.28 nm	f = 0.2276
	H−1 → L+1	-0.22271
	H → L	0.66060
S_0–S_3	306.15 nm	f = 0.0268
S_0–S_4	299.49 nm	f = 0.2322
S_0–S_5	298.99 nm	f = 0.0765
S_0–S_6	287.96 nm	f = 0.3767
S_0–S_7	272.34 nm	f = 0.0733
S_0–S_8	262.34 nm	f = 0.4003
S_0–S_9	259.90 nm	f = 0.7874
S_0–S_{10}	251.74 nm	f = 0.0019
S_0–S_{11}	250.96 nm	f = 0.0005
S_0–S_{12}	250.26 nm	f = 0.0398
S_0–S_{13}	249.73 nm	f = 0.0000
S_0–S_{14}	243.46 nm	f = 0.0138
S_0–S_{15}	241.20 nm	f = 0.4809
S_0–S_{16}	240.63 nm	f = 0.0006
S_0–S_{17}	233.23 nm	f = 0.0000
S_0–S_{18}	231.95 nm	f = 0.0025
S_0–S_{19}	228.76 nm	f = 0.0000
S_0–S_{20}	226.85 nm	f = 0.0146
S_0–S_{21}	221.99 nm	f = 0.0059
S_0–S_{22}	221.20 nm	f = 0.0025
S_0–S_{23}	221.07 nm	f = 0.0438
S_0–S_{24}	217.06 nm	f = 0.0075
S_0–S_{25}	214.37 nm	f = 0.0122

(e) F_8FUL

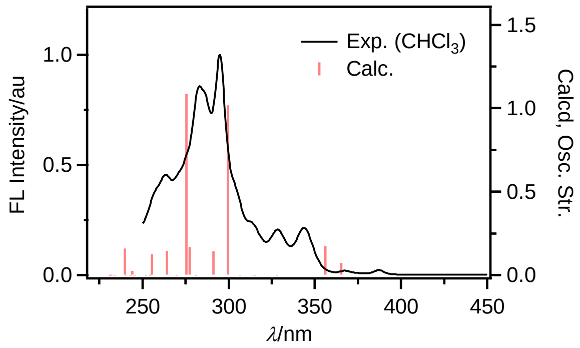

S_0–S_1	365.25 nm	f = 0.0731
	H−1 → L	-0.24411
	H−1 → L+1	-0.16549
	H → L	0.55583
	H → L+1	-0.30881
S_0–S_2	356.07 nm	f = 0.1742
	H−1 → L+1	0.29571
	H → L	0.38598
	H → L+1	0.49913
S_0–S_3	328.04 nm	f = 0.0000
S_0–S_4	315.05 nm	f = 0.0000
S_0–S_5	306.60 nm	f = 0.0000

S_0–S_6	299.61 nm	$f = 1.0189$
S_0–S_7	291.22 nm	$f = 0.1419$
S_0–S_8	280.82 nm	$f = 0.0000$
S_0–S_9	277.45 nm	$f = 0.1684$
S_0–S_{10}	275.58 nm	$f = 1.0849$
S_0–S_{11}	269.86 nm	$f = 0.0000$
S_0–S_{12}	264.08 nm	$f = 0.1453$
S_0–S_{13}	255.40 nm	$f = 0.1258$
S_0–S_{14}	254.84 nm	$f = 0.0019$
S_0–S_{15}	254.28 nm	$f = 0.0000$
S_0–S_{16}	252.00 nm	$f = 0.0000$
S_0–S_{17}	245.37 nm	$f = 0.0000$
S_0–S_{18}	244.04 nm	$f = 0.0238$
S_0–S_{19}	242.90 nm	$f = 0.0000$
S_0–S_{20}	240.40 nm	$f = 0.0000$
S_0–S_{21}	239.76 nm	$f = 0.1584$
S_0–S_{22}	234.20 nm	$f = 0.0000$
S_0–S_{23}	231.95 nm	$f = 0.0041$
S_0–S_{24}	231.34 nm	$f = 0.0000$
S_0–S_{25}	231.15 nm	$f = 0.0000$

(f) $F_8$7PHEN

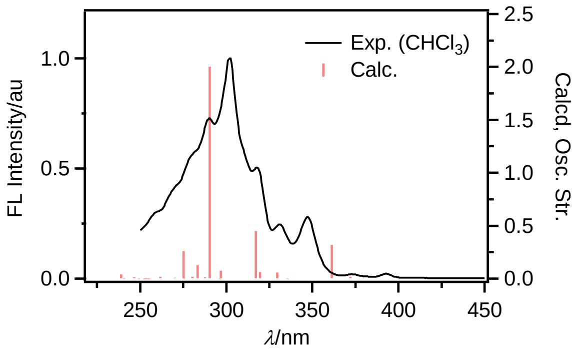

S_0–S_1	372.01 nm	$f = 0.0160$
H−2 → L	-0.16647	
H−1 → L	0.29756	
H → L+1	0.59555	
H → L+2	0.11682	
S_0–S_2	361.25 nm	$f = 0.3188$
H−2 → L+1	0.10053	
H−1 → L+1	-0.19164	
H → L	0.66671	
S_0–S_3	335.62 nm	$f = 0.0007$
S_0–S_4	329.61 nm	$f = 0.0583$
S_0–S_5	319.67 nm	$f = 0.0616$
S_0–S_6	317.01 nm	$f = 0.4509$
S_0–S_7	296.79 nm	$f = 0.0760$
S_0–S_8	296.44 nm	$f = 0.0000$
S_0–S_9	290.47 nm	$f = 2.0034$
S_0–S_{10}	287.64 nm	$f = 0.0130$
S_0–S_{11}	283.19 nm	$f = 0.1269$
S_0–S_{12}	280.44 nm	$f = 0.0186$
S_0–S_{13}	275.57 nm	$f = 0.0034$
S_0–S_{14}	275.17 nm	$f = 0.2612$
S_0–S_{15}	270.10 nm	$f = 0.0095$
S_0–S_{16}	261.57 nm	$f = 0.0165$
S_0–S_{17}	255.05 nm	$f = 0.0022$
S_0–S_{18}	254.74 nm	$f = 0.0000$
S_0–S_{19}	253.54 nm	$f = 0.0060$
S_0–S_{20}	252.89 nm	$f = 0.0003$
S_0–S_{21}	252.23 nm	$f = 0.0018$
S_0–S_{22}	249.16 nm	$f = 0.0001$
S_0–S_{23}	246.51 nm	$f = 0.0124$
S_0–S_{24}	240.63 nm	$f = 0.0033$
S_0–S_{25}	238.93 nm	$f = 0.0418$

4. NMR spectra of the synthesized compounds.

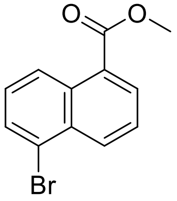

1

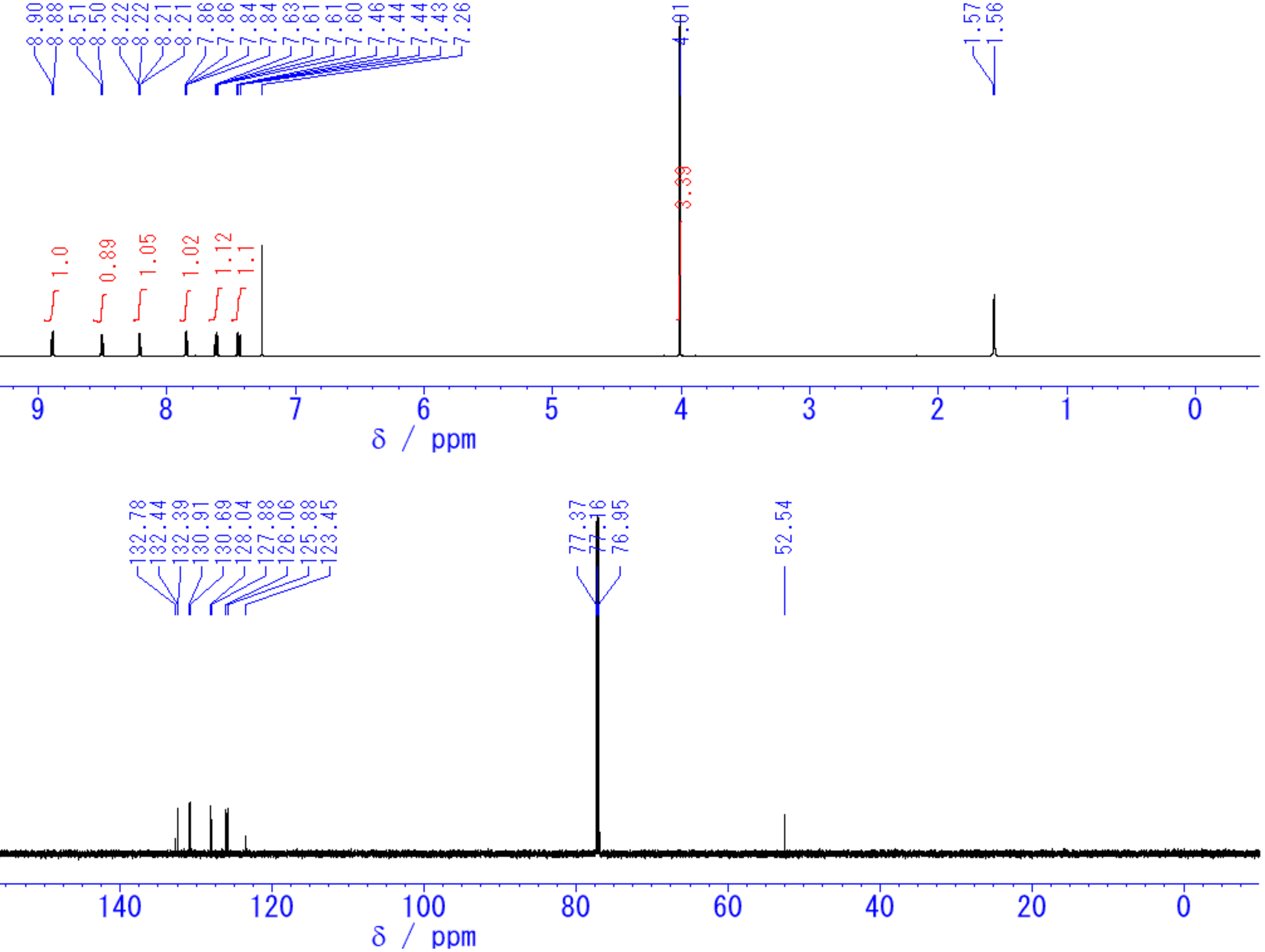

^{1}H (600 MHz, upper) and ^{13}C (151 MHz, lower) NMR spectra of compound **1** in $CDCl_3$.

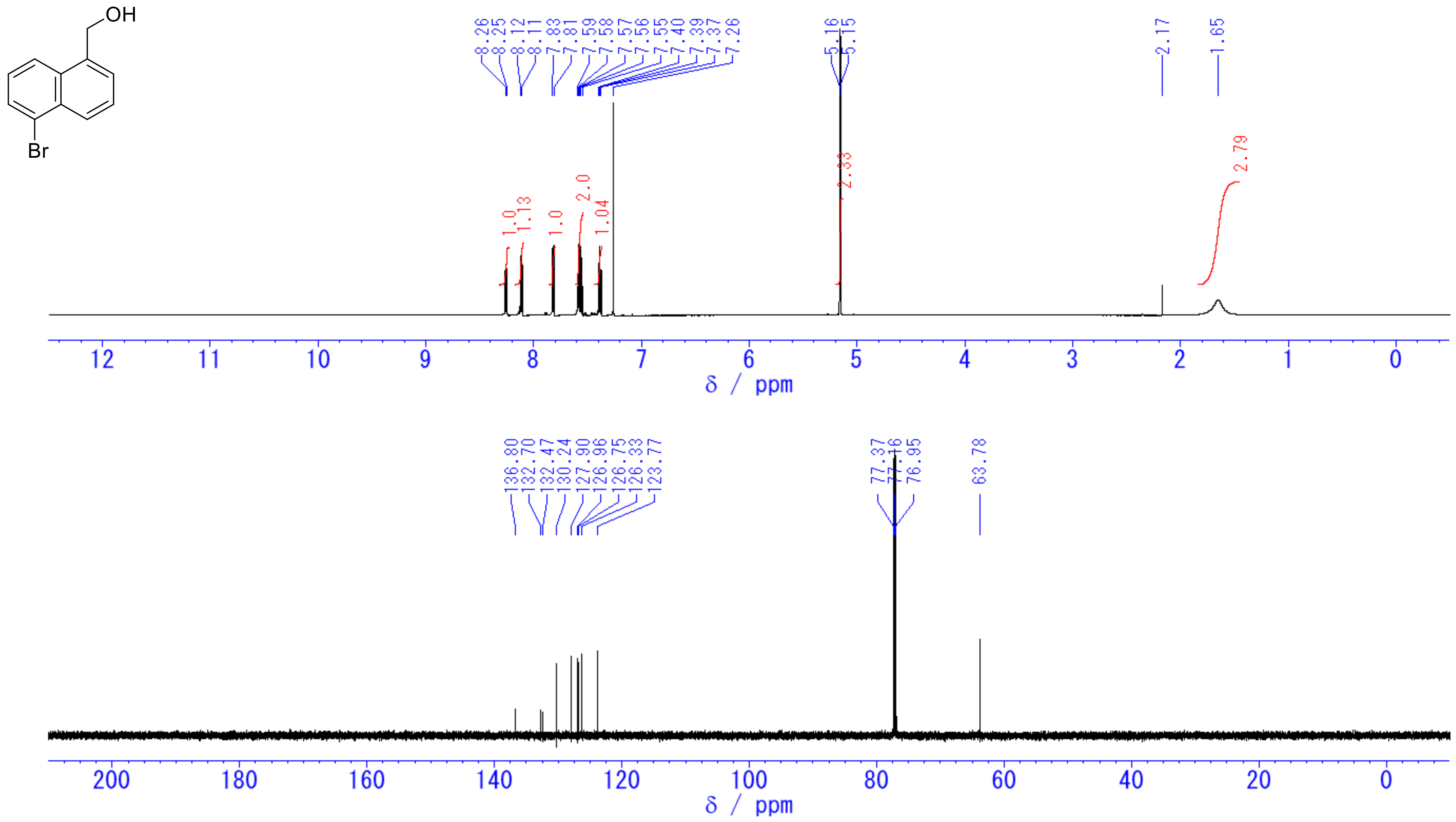

^{1}H (600 MHz, upper) and ^{13}C (151 MHz, lower) NMR spectra of 5-bromo-1-naphthalenemethanol in $CDCl_3$.

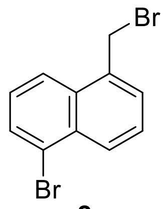

2

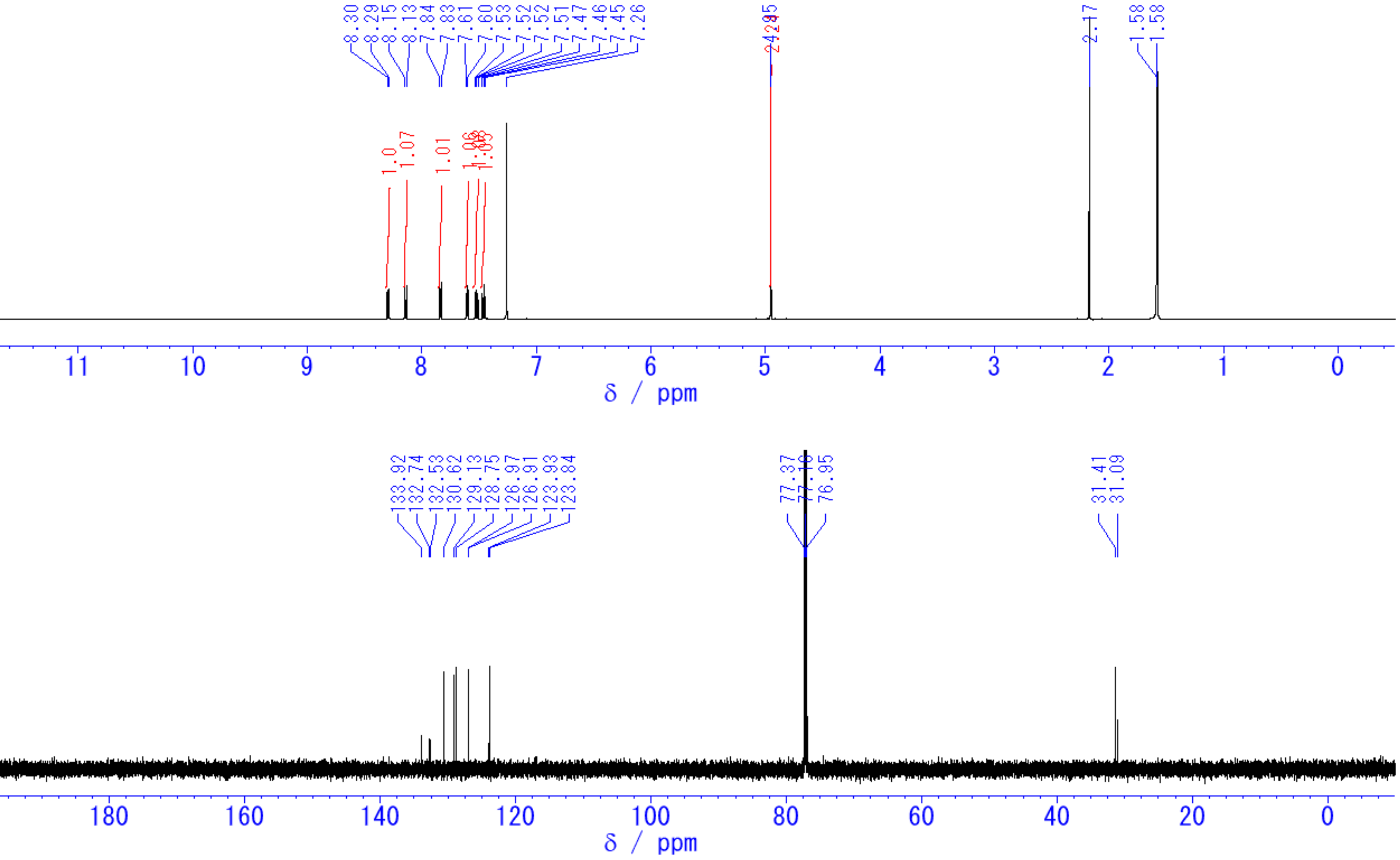

^1H (600 MHz, upper) and ^{13}C (151 MHz, lower) NMR spectra of compound **2** in $CDCl_3$.

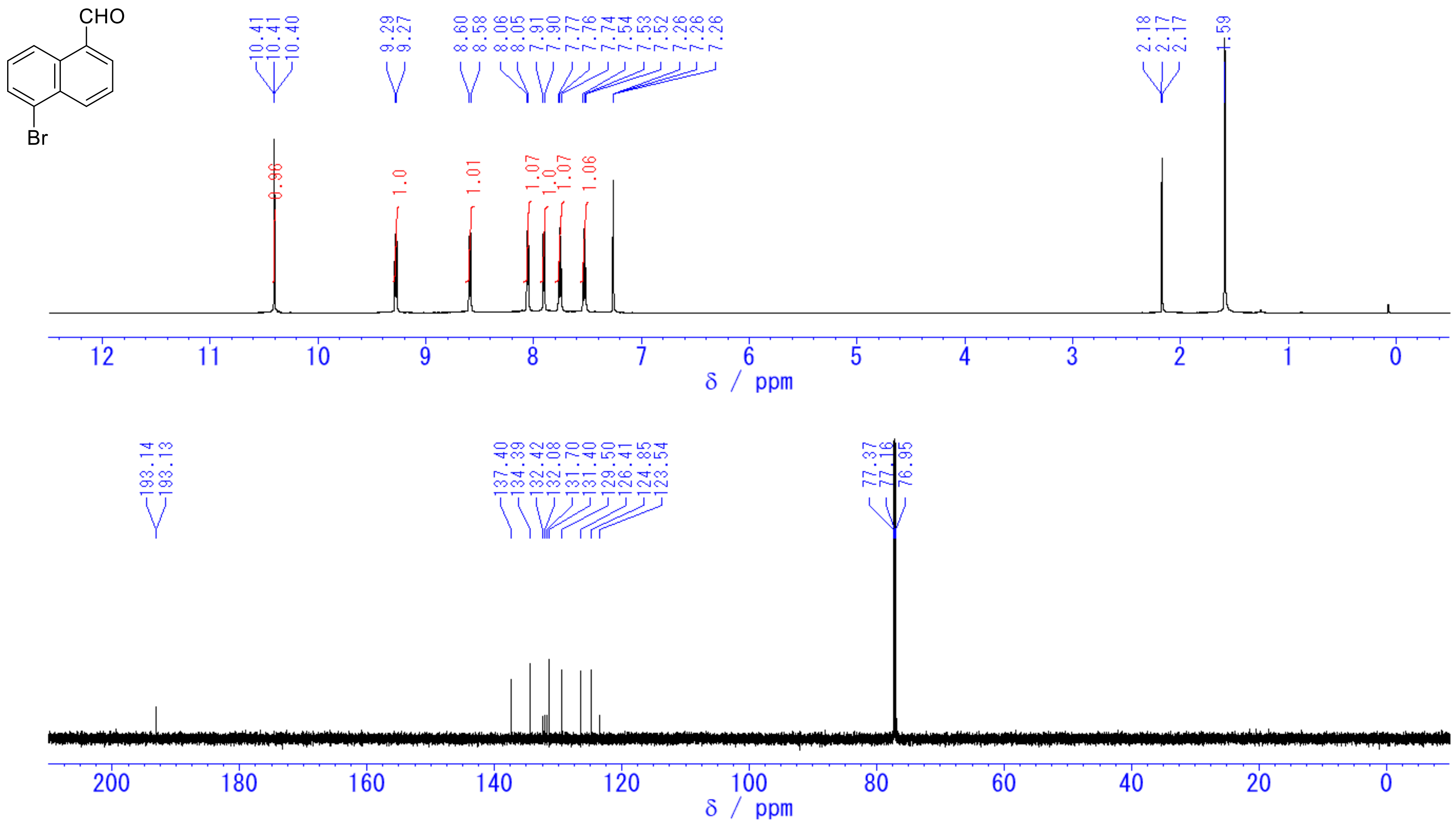

^{1}H (600 MHz, upper) and ^{13}C (151 MHz, lower) NMR spectra of 5-bromo-1-naphthaldehyde in $CDCl_3$.

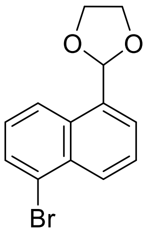

3

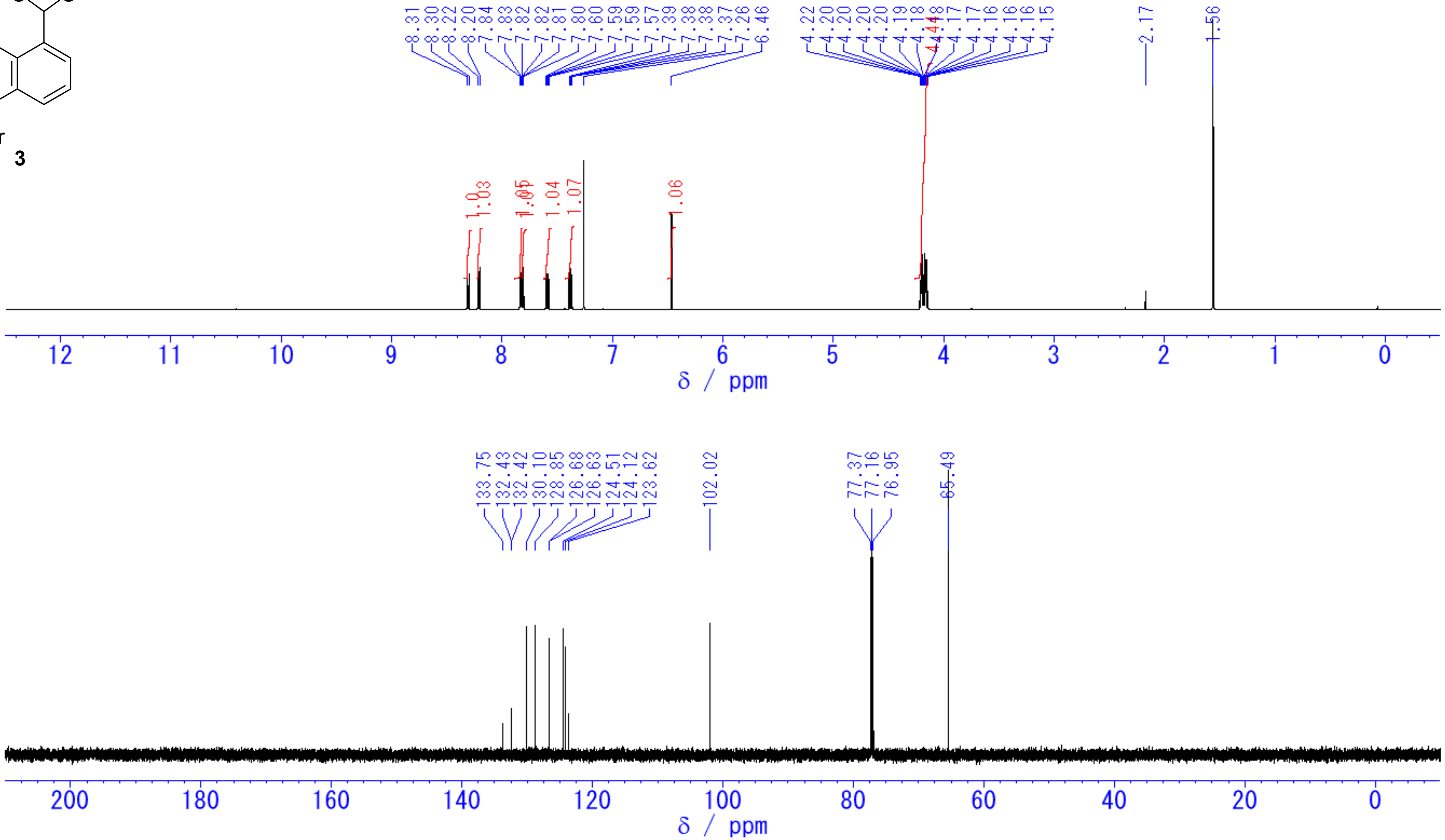

^{1}H (600 MHz, upper) and ^{13}C (151 MHz, lower) NMR spectra of compound **3** in $CDCl_3$.

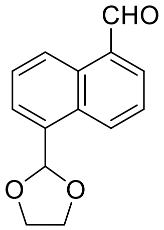

4

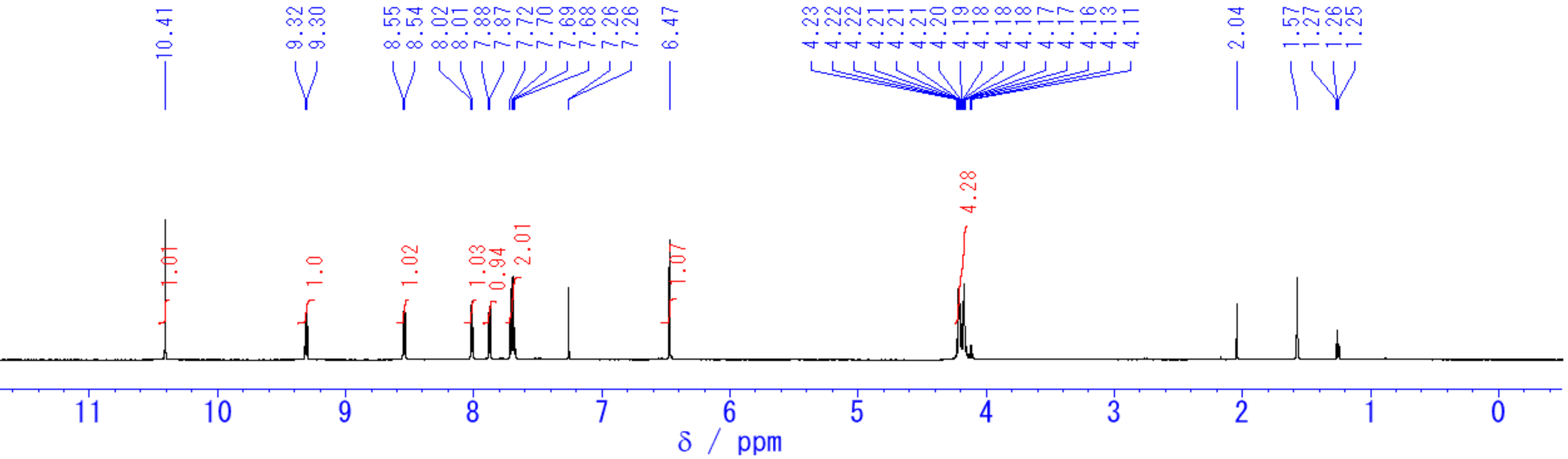

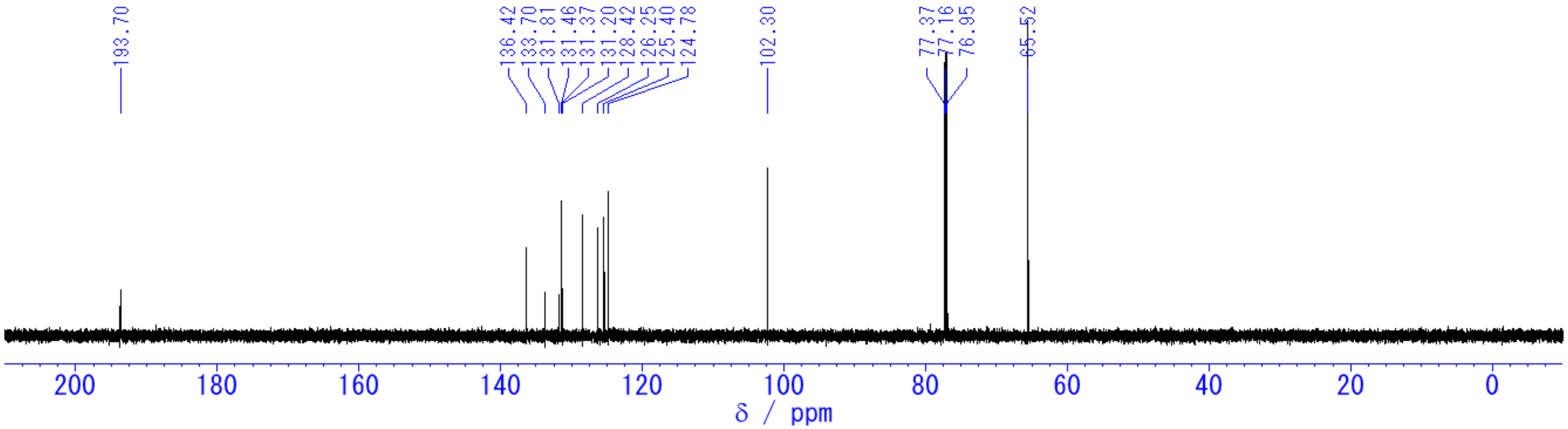

^{1}H (600 MHz, upper) and ^{13}C (151 MHz, lower) NMR spectra of compound **4** in $CDCl_3$.

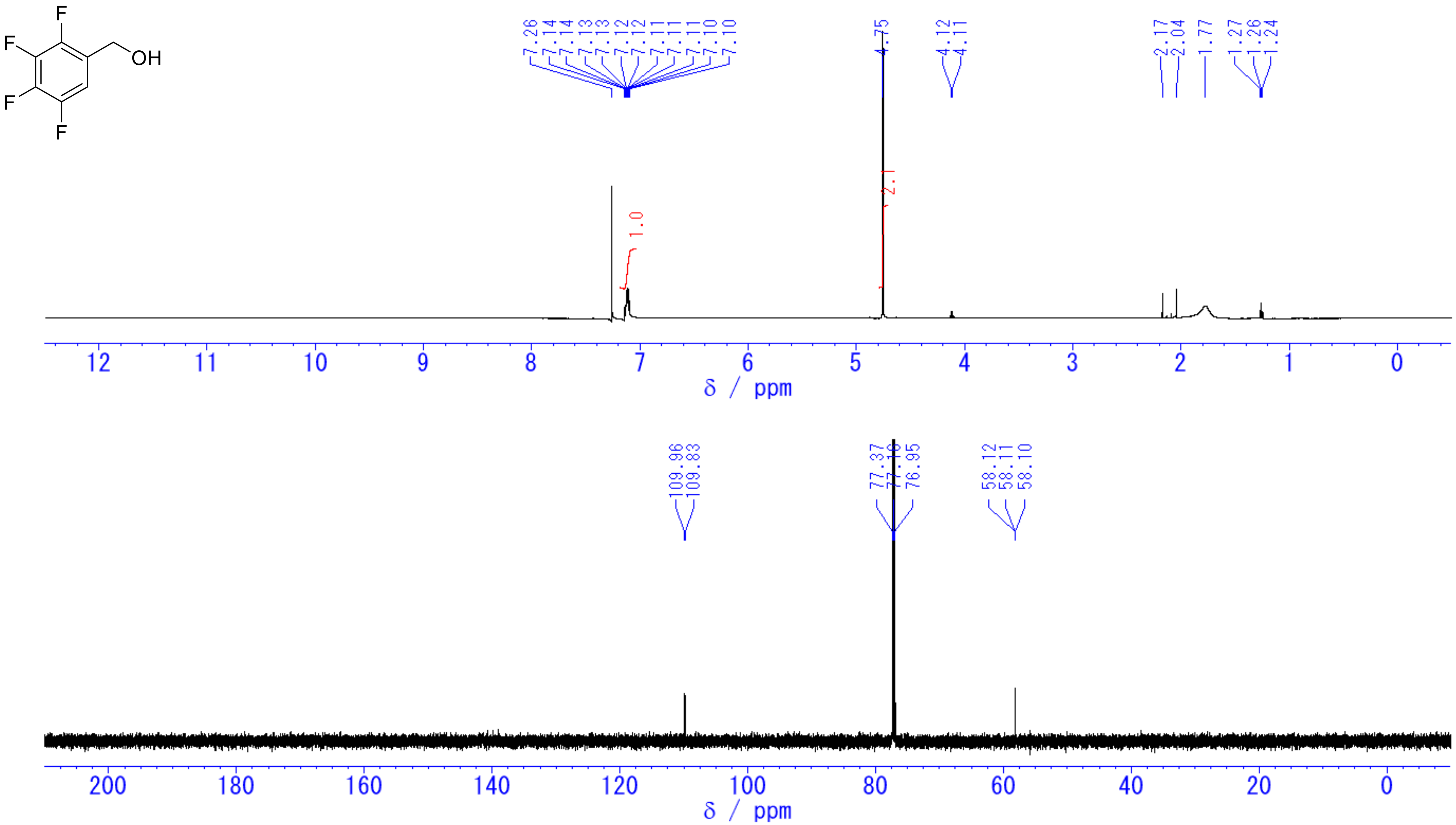

^{1}H (600 MHz, upper) and ^{13}C (151 MHz, lower) NMR spectra of 2,3,4,5-tetrafluorobenzyl alcohol in $CDCl_3$.

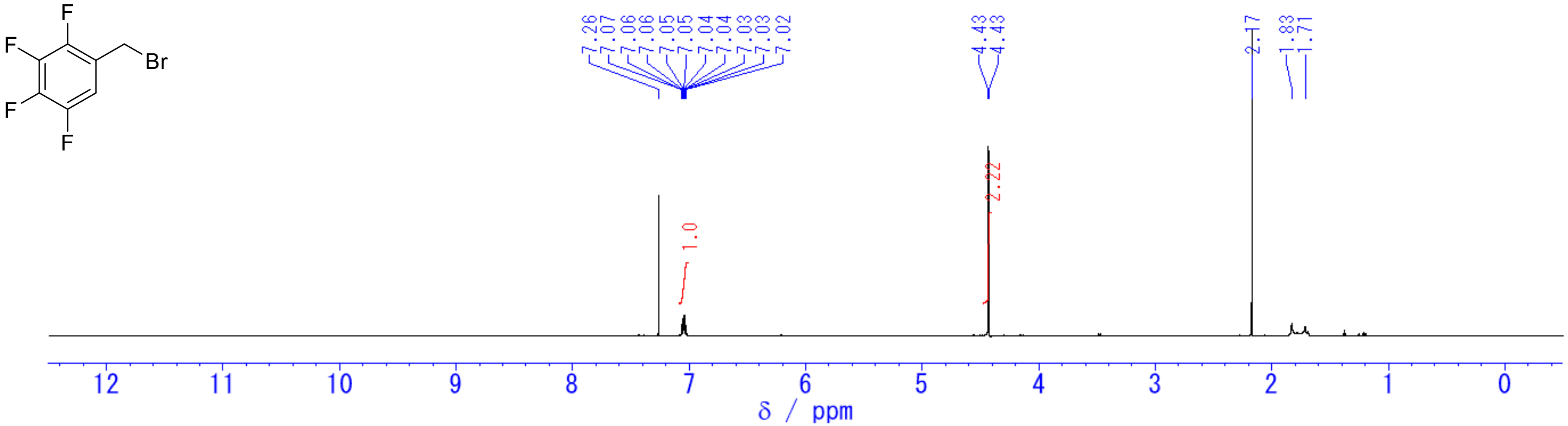

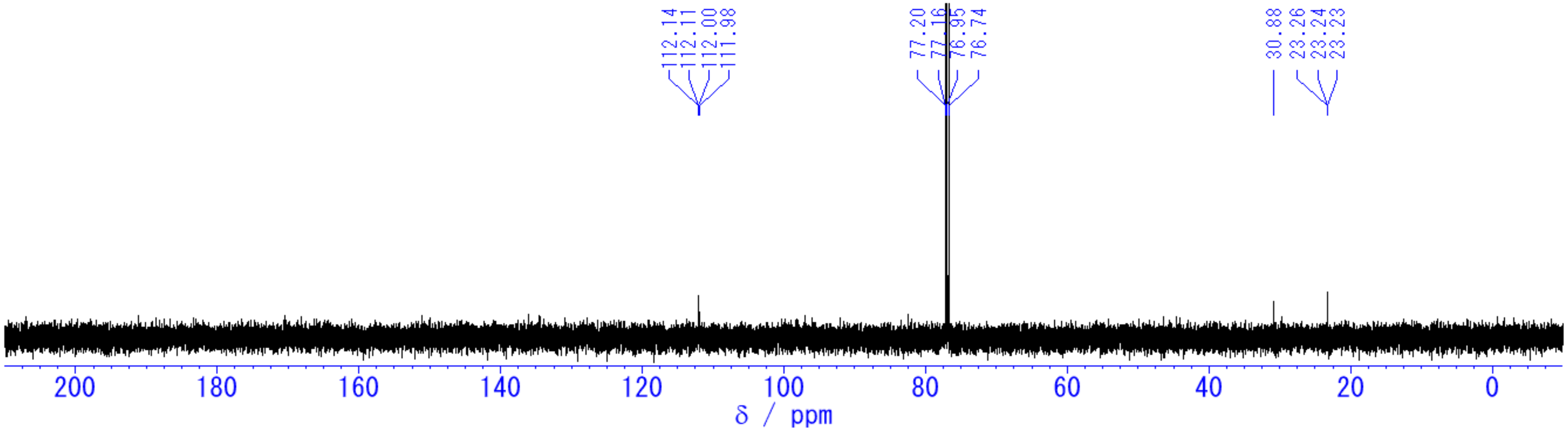

^{1}H (600 MHz, upper) and ^{13}C (151 MHz, lower) NMR spectra of 2,3,4,5-tetrafluorobenzyl bromide in $CDCl_3$.

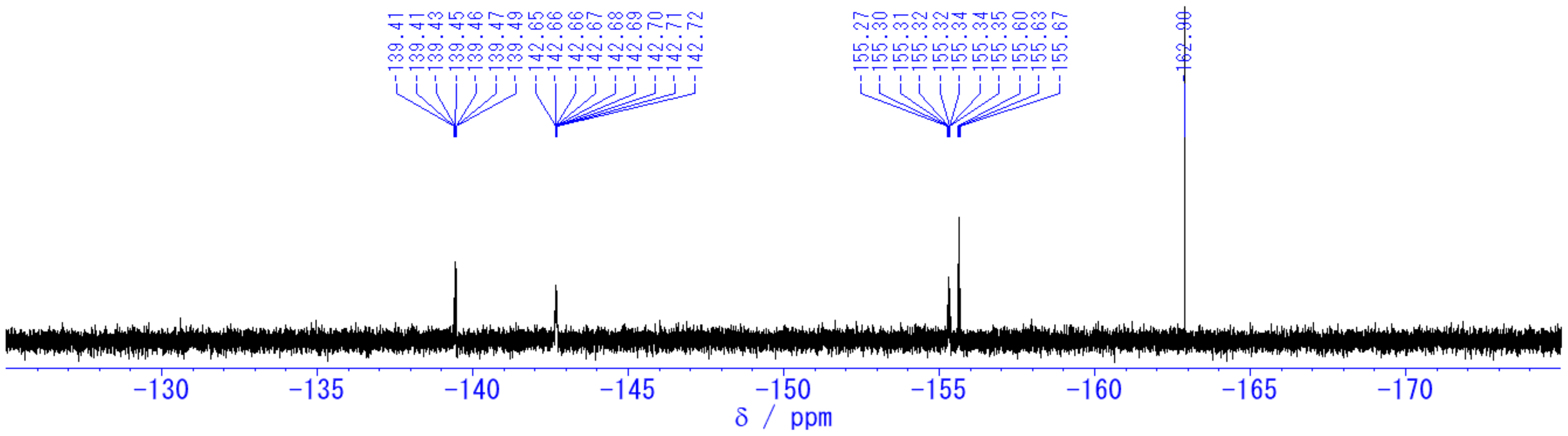

^{19}F (564 MHz) NMR spectrum of compound 2,3,4,5-tetrafluorobenzyl bromide in $CDCl_3$.

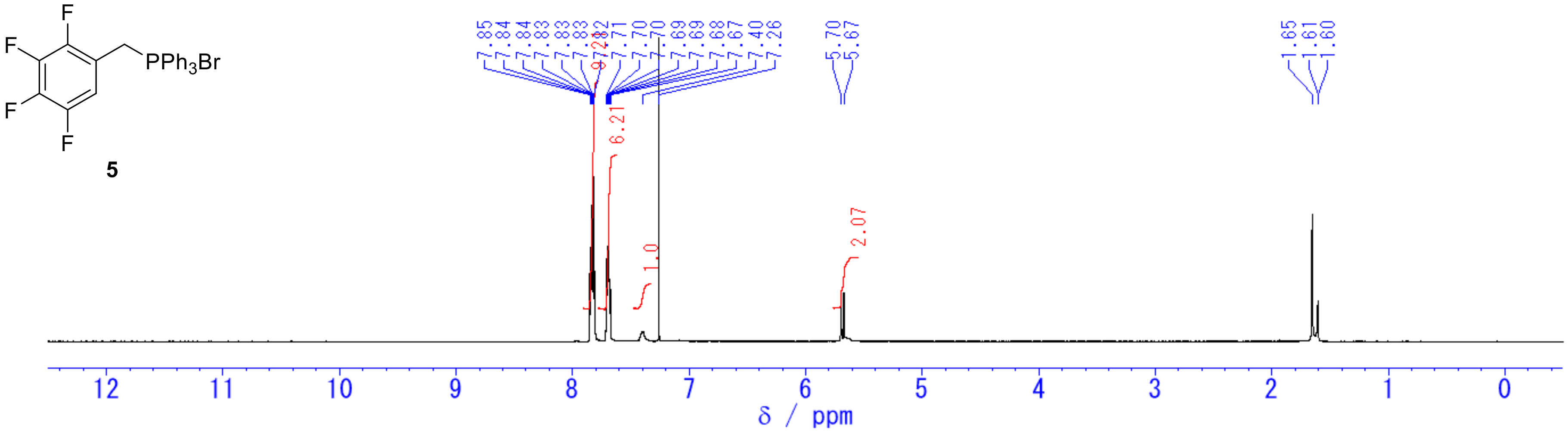

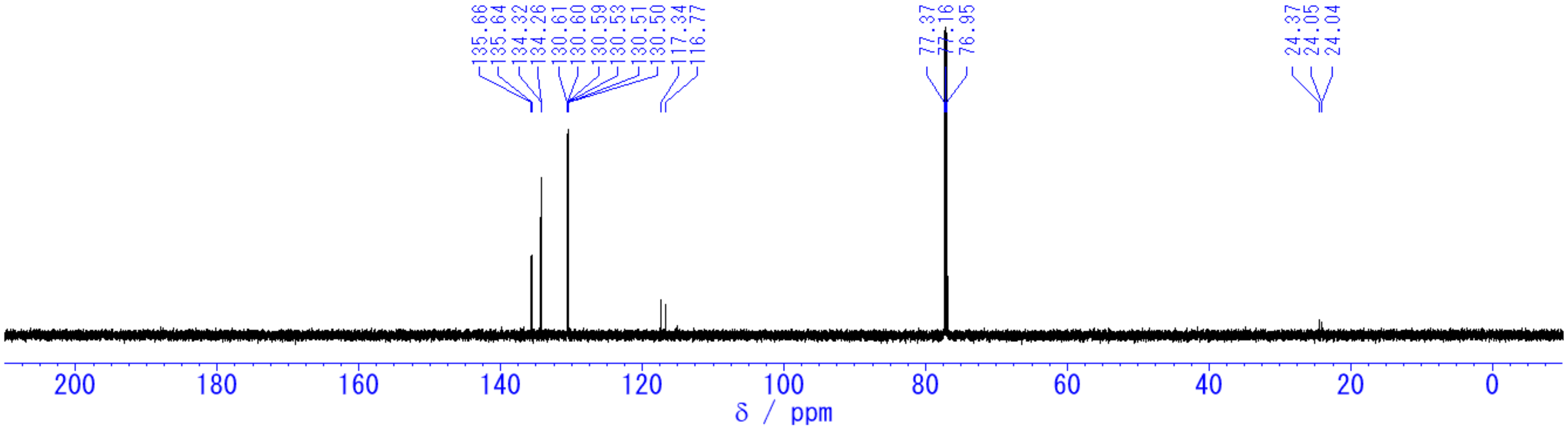

^{1}H (600 MHz, upper) and ^{13}C (151 MHz, lower) NMR spectra of compound **5** in $CDCl_3$.

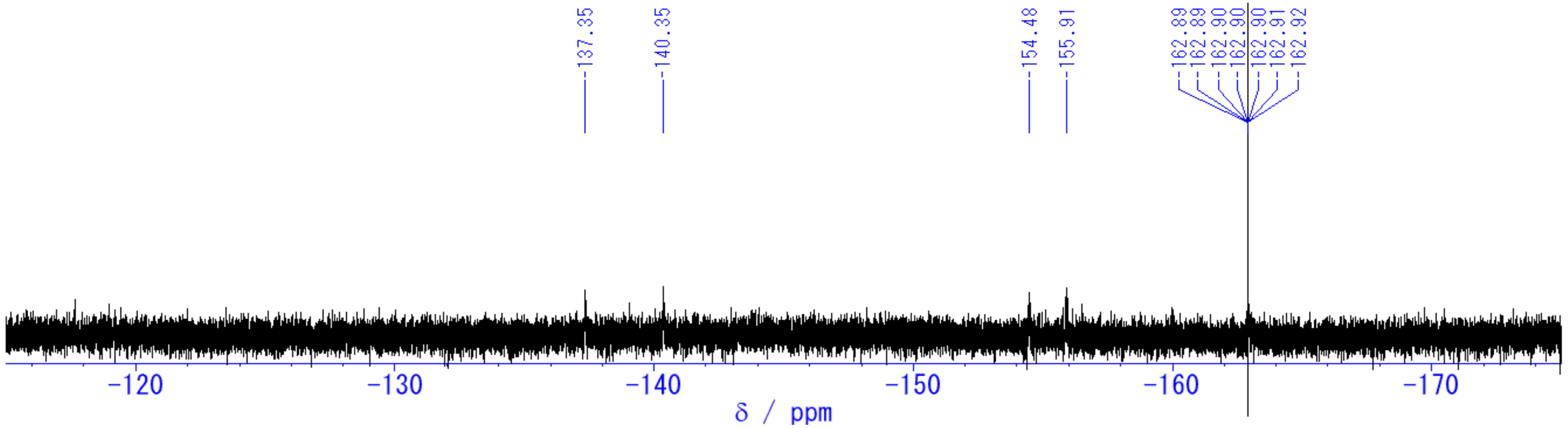

^{19}F (564 MHz) NMR spectrum of compound **5** in $CDCl_3$.

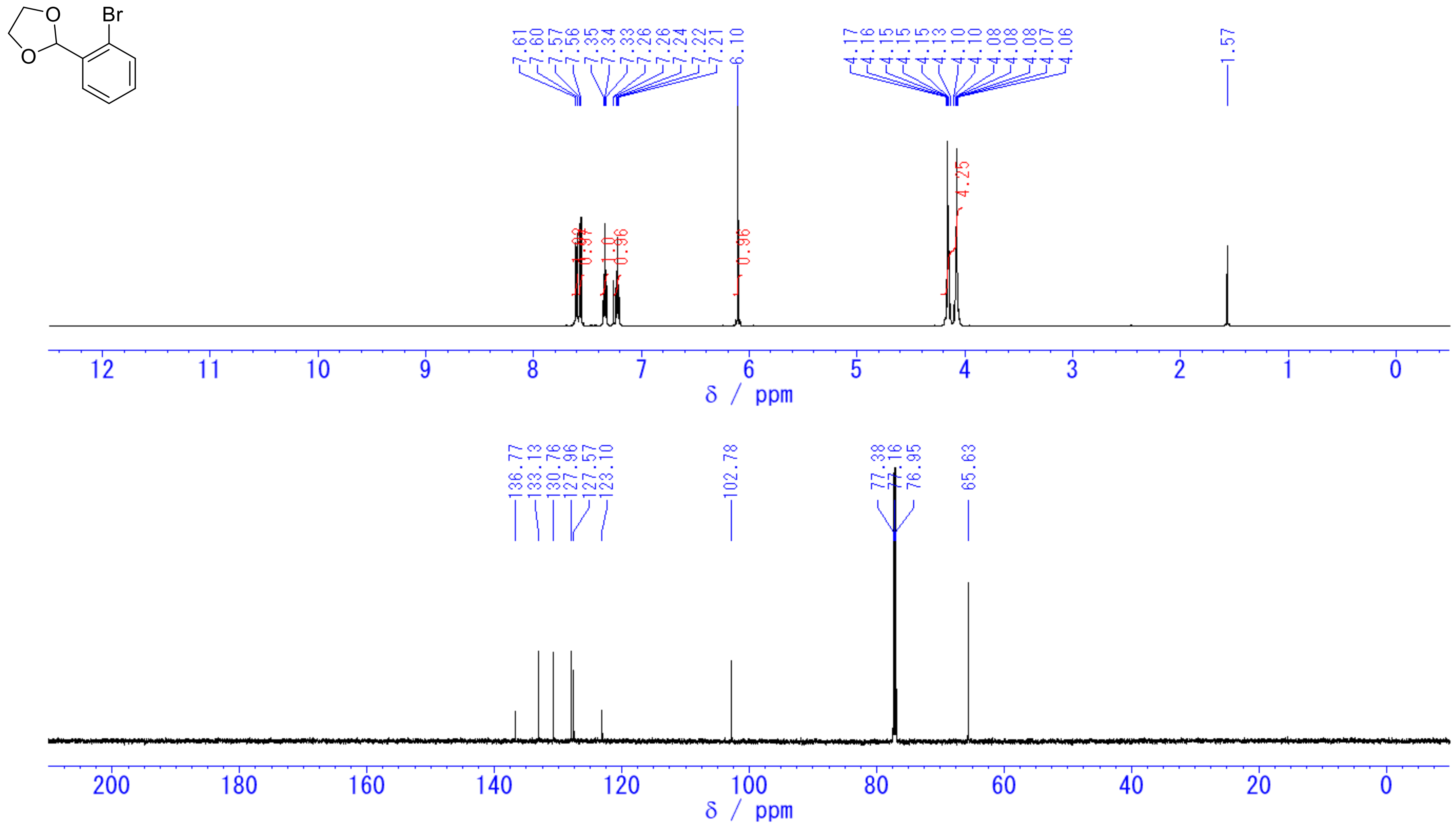

^{1}H (600 MHz, upper) and ^{13}C (151 MHz, lower) NMR spectra of 2-(2-bromophenyl)-1,3-dioxolane in $CDCl_3$.

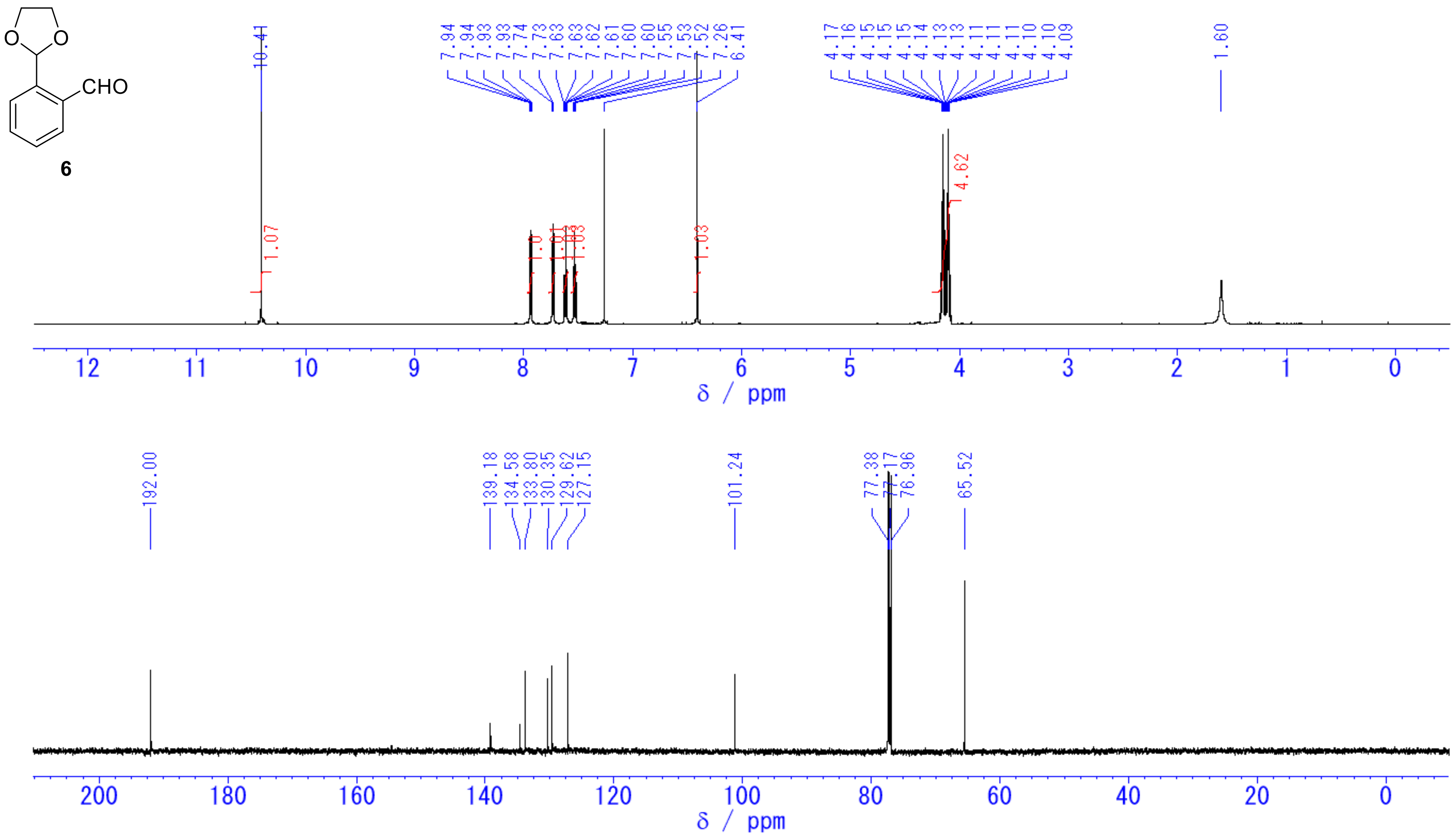

^{1}H (600 MHz, upper) and ^{13}C (151 MHz, lower) NMR spectra of compound **6** in $CDCl_3$.

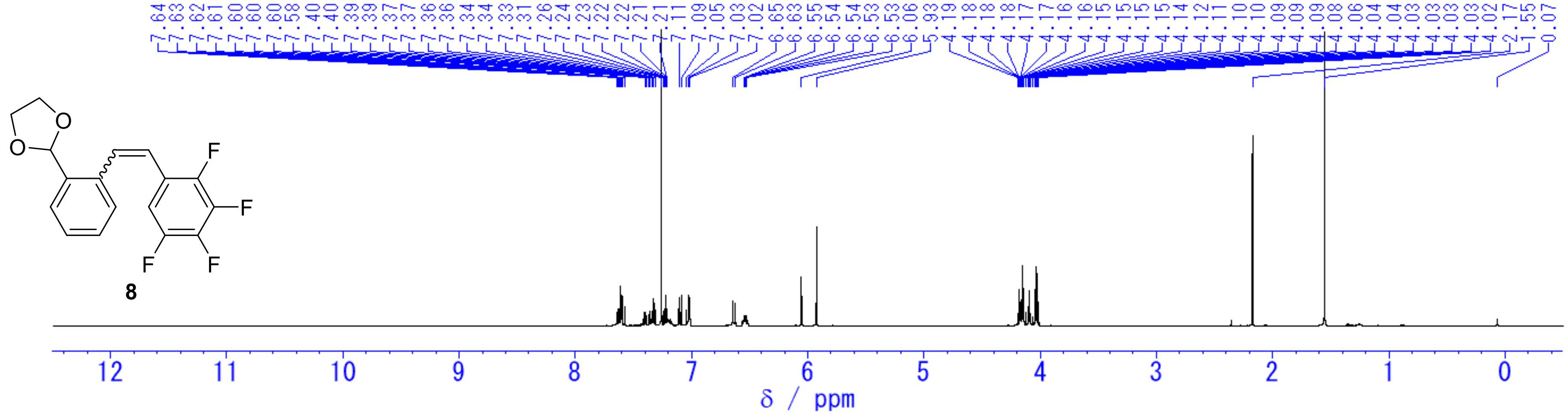

^{1}H (600 MHz) NMR spectrum of compound **8** in $CDCl_3$.

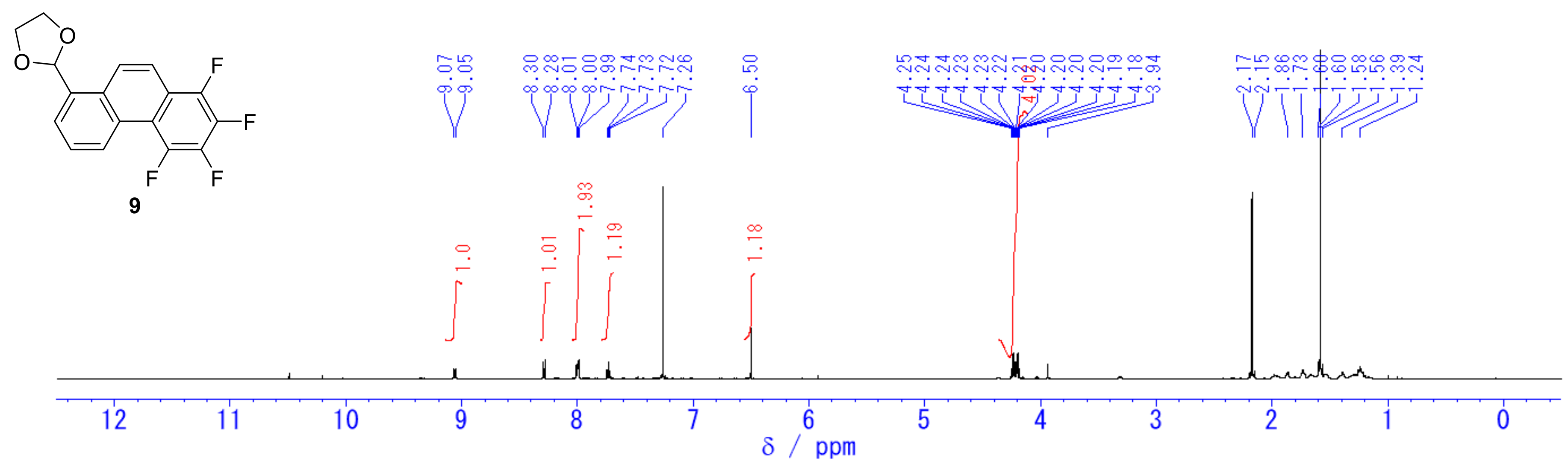

^{1}H (600 MHz) NMR spectrum of compound **9** in $CDCl_3$.

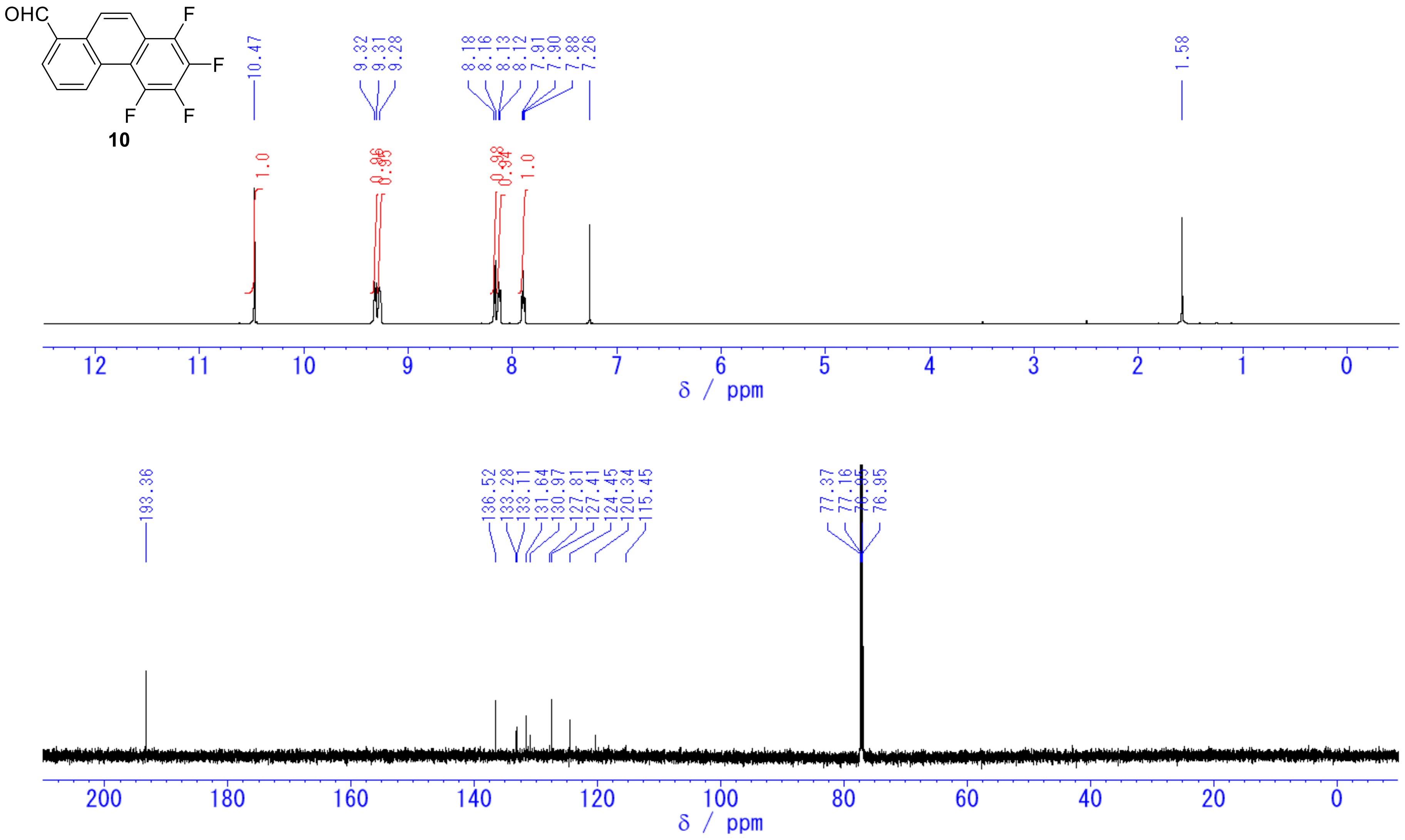

^{1}H (600 MHz, upper) and ^{13}C (151 MHz, lower) NMR spectra of compound **10** in $CDCl_3$.

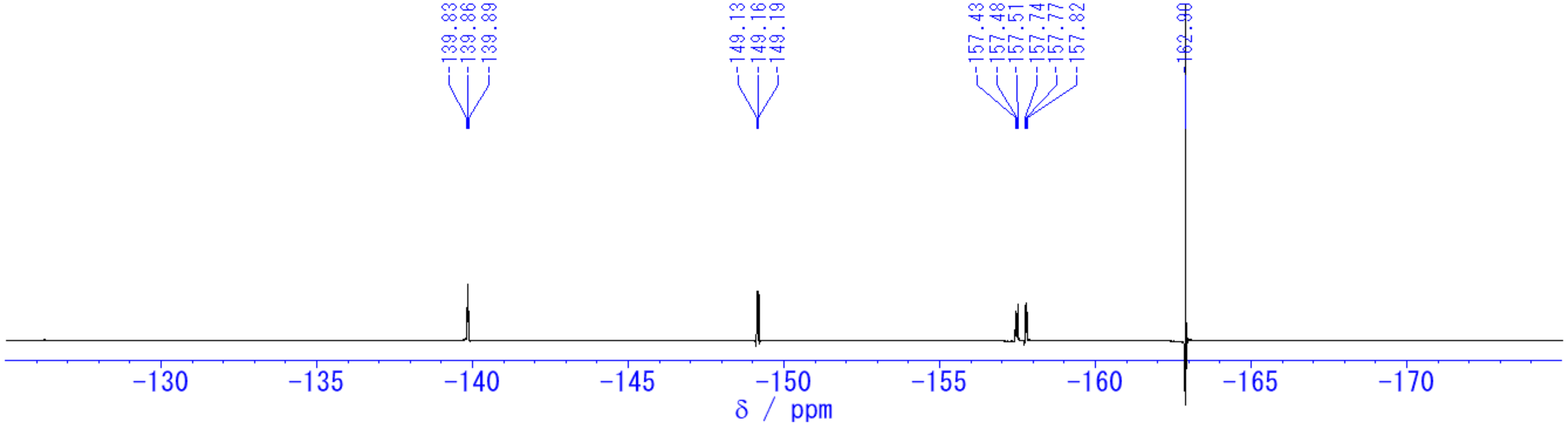

^{19}F (564 MHz) NMR spectrum of compound **10** in $CDCl_3$.

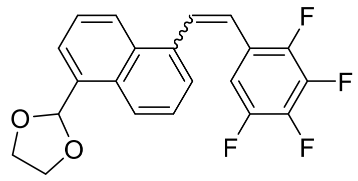

11

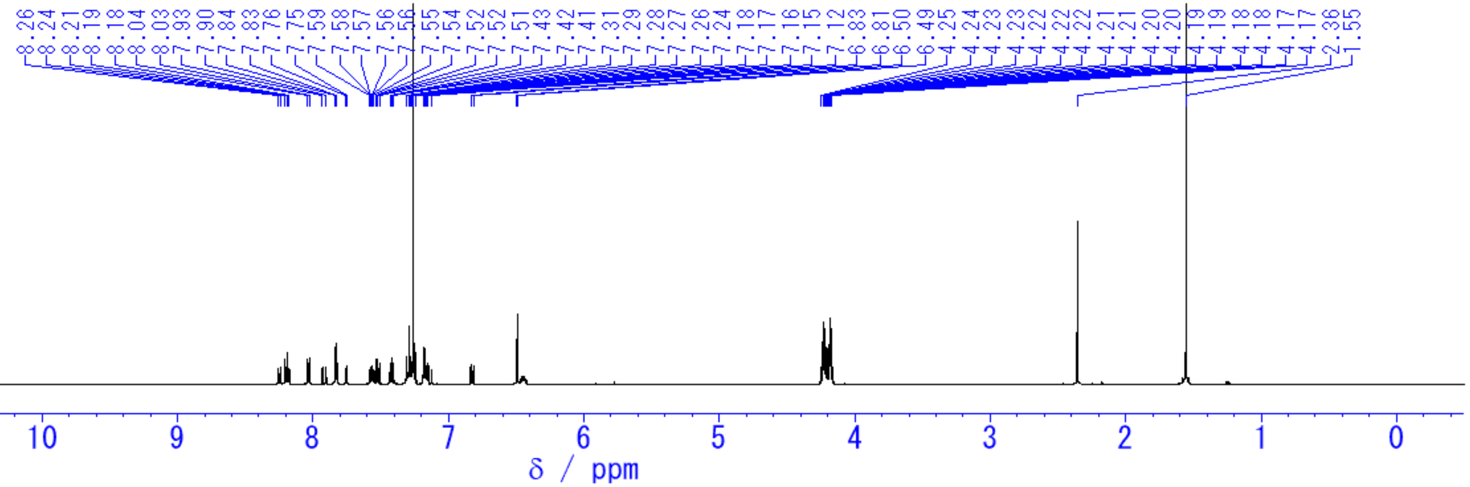

^{1}H (600 MHz) NMR spectrum of compound **11** in $CDCl_3$.

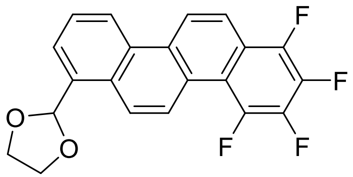

12

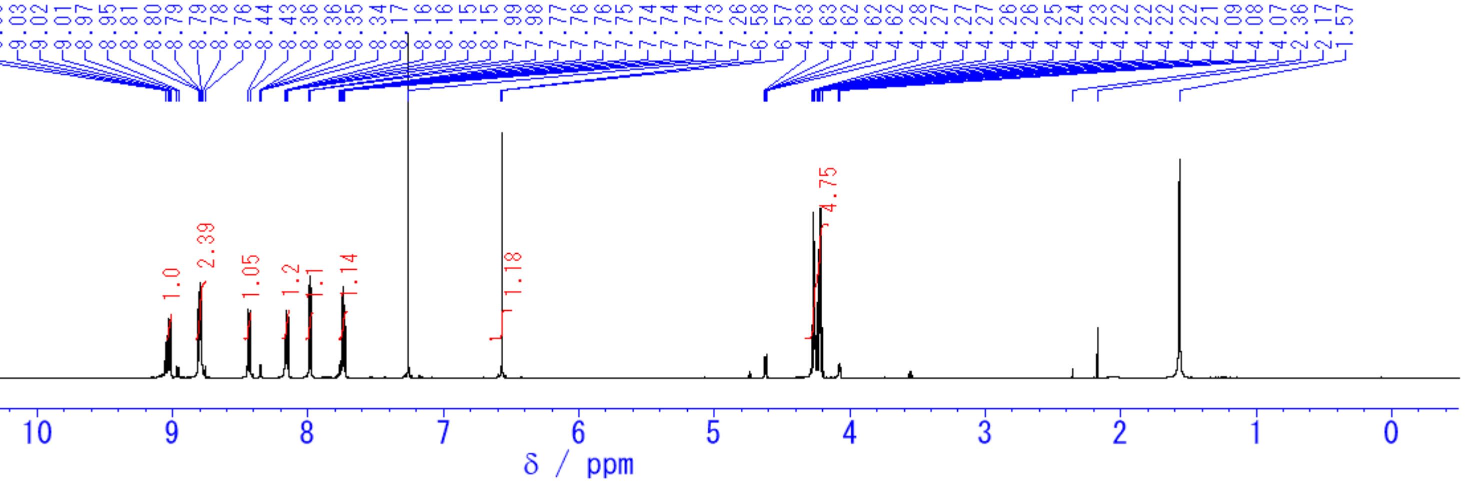

^{1}H (600 MHz) NMR spectrum of compound **12** in $CDCl_3$.

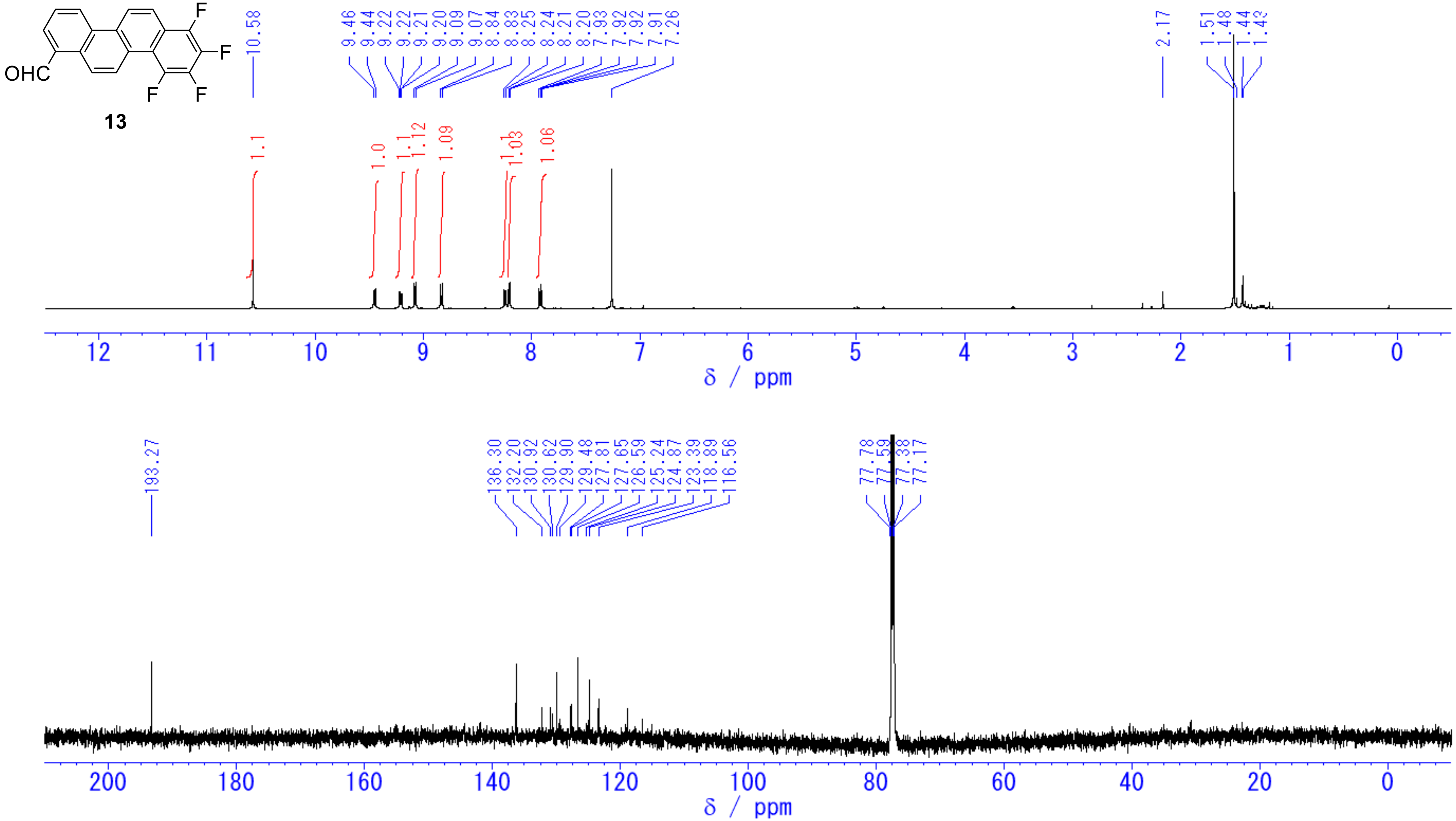

^{1}H (600 MHz, upper) and ^{13}C (151 MHz, lower) NMR spectra of compound **13** in $CDCl_3$.

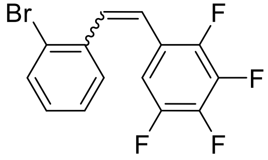

14

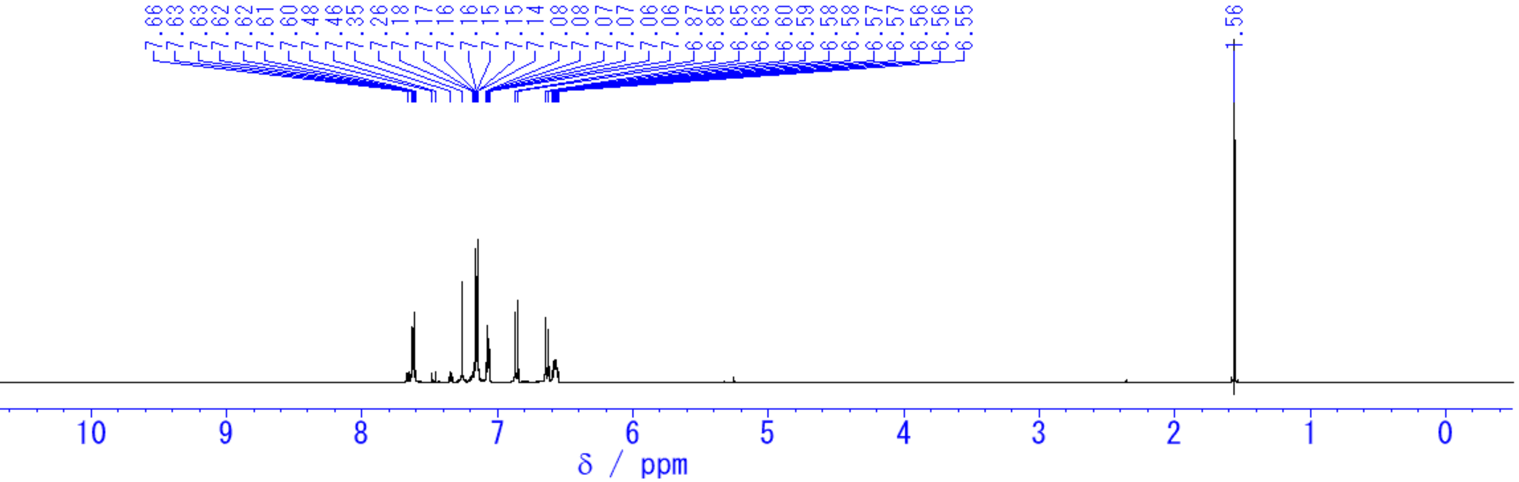

^{1}H (600 MHz) NMR spectrum of compound **14** in $CDCl_3$.

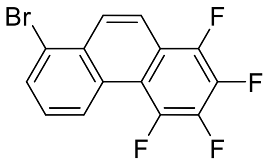

15

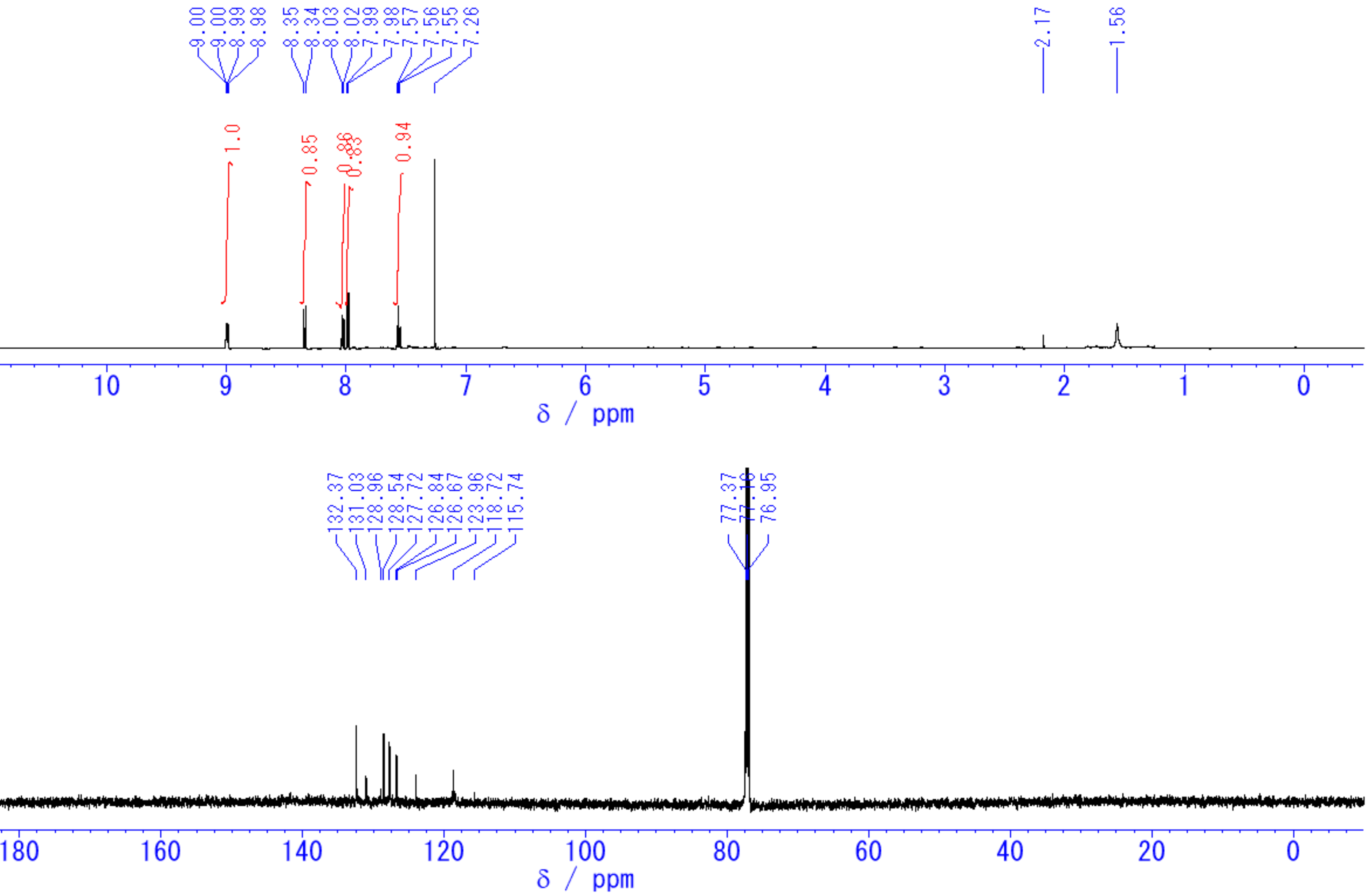

^{1}H (600 MHz, upper) and ^{13}C (151 MHz, lower) NMR spectra of compound **15** in $CDCl_3$.

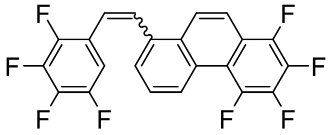

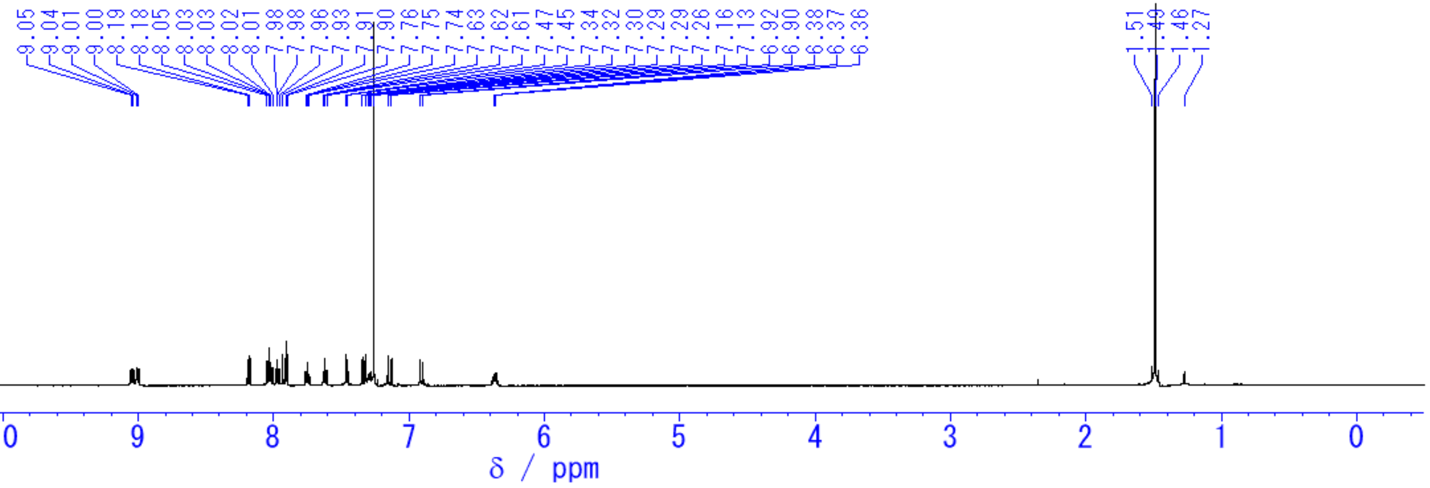

^{1}H (600 MHz) NMR spectrum of compound **16** in $CDCl_3$.

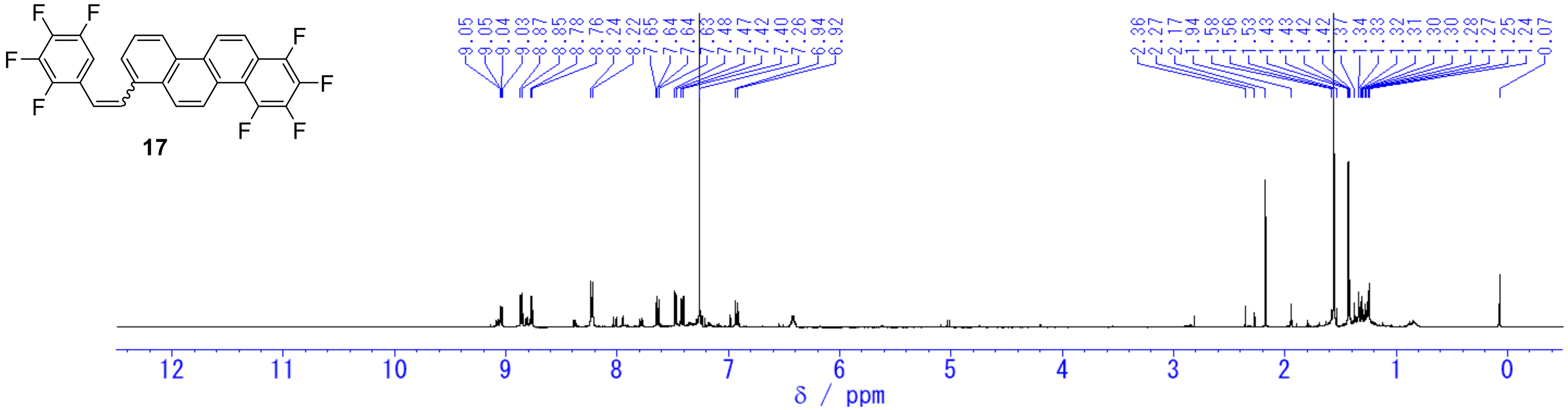

^{1}H (600 MHz) NMR spectrum of compound **17** in $CDCl_3$.

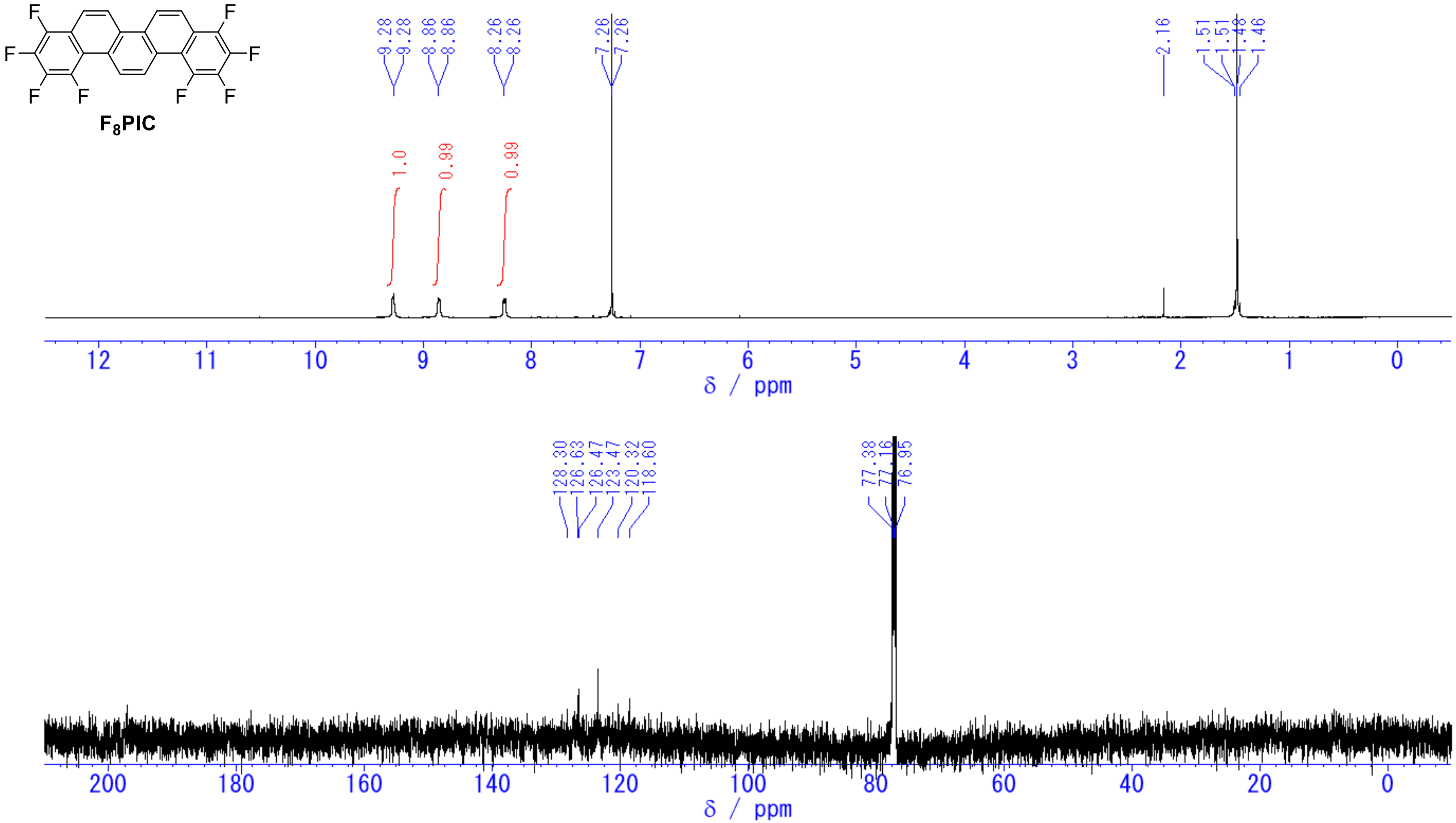

^{1}H (600 MHz, upper) and ^{13}C (151 MHz, lower) NMR spectra of **F8PIC** in $CDCl_3$ (50 °C).

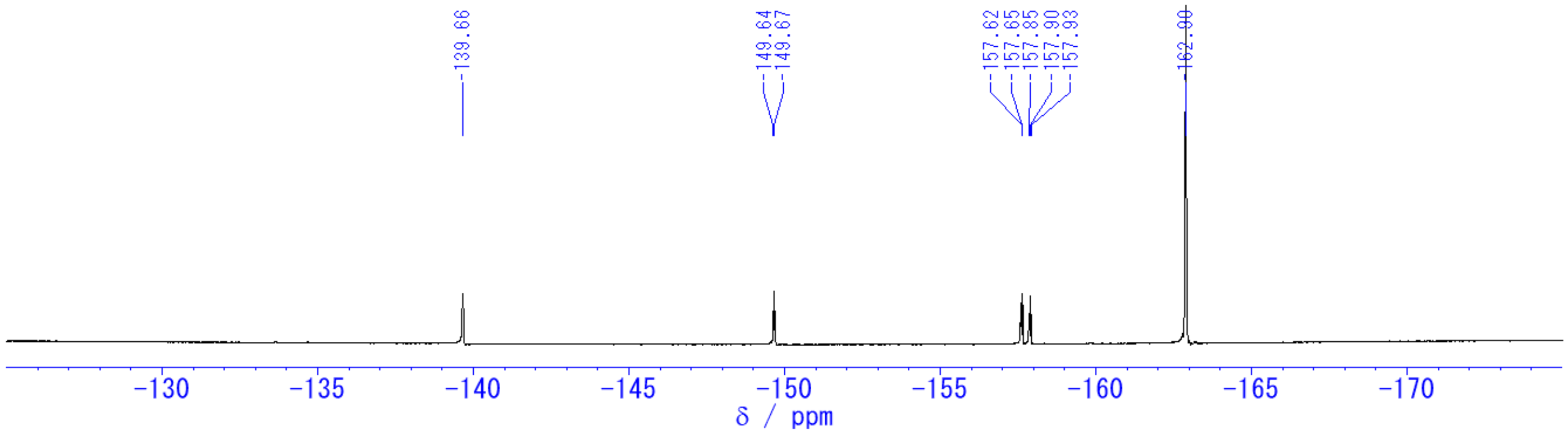

^{19}F (564 MHz) NMR spectrum of **F8PIC** in $CDCl_3$ (50 °C).

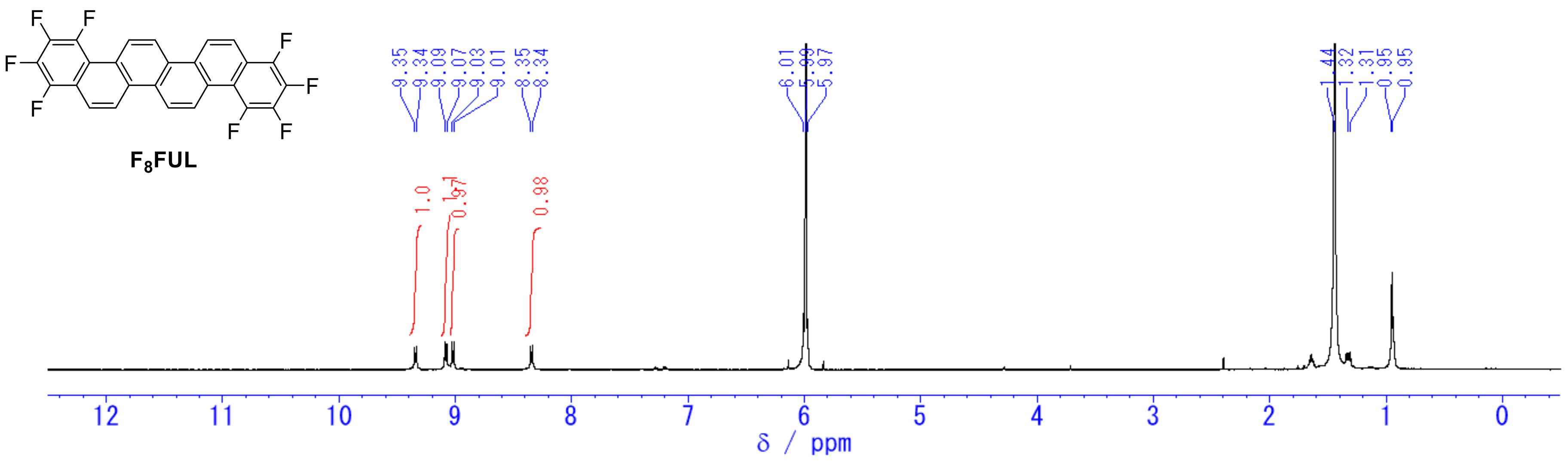

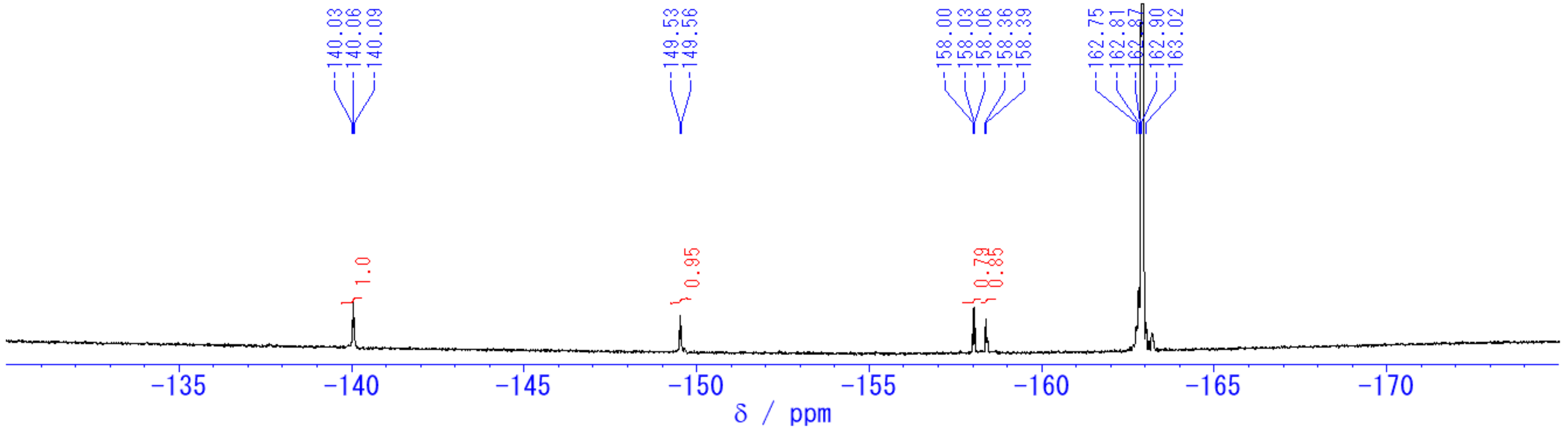

^{1}H (600 MHz, upper) and ^{19}F (564 MHz, lower) NMR spectra of **F8FUL** in 1,1,2,2-tetrachloroethane-d_2 at 120 °C.

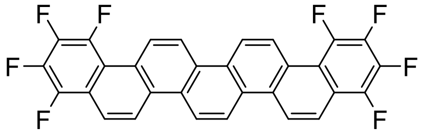

$F_8$7PHEN

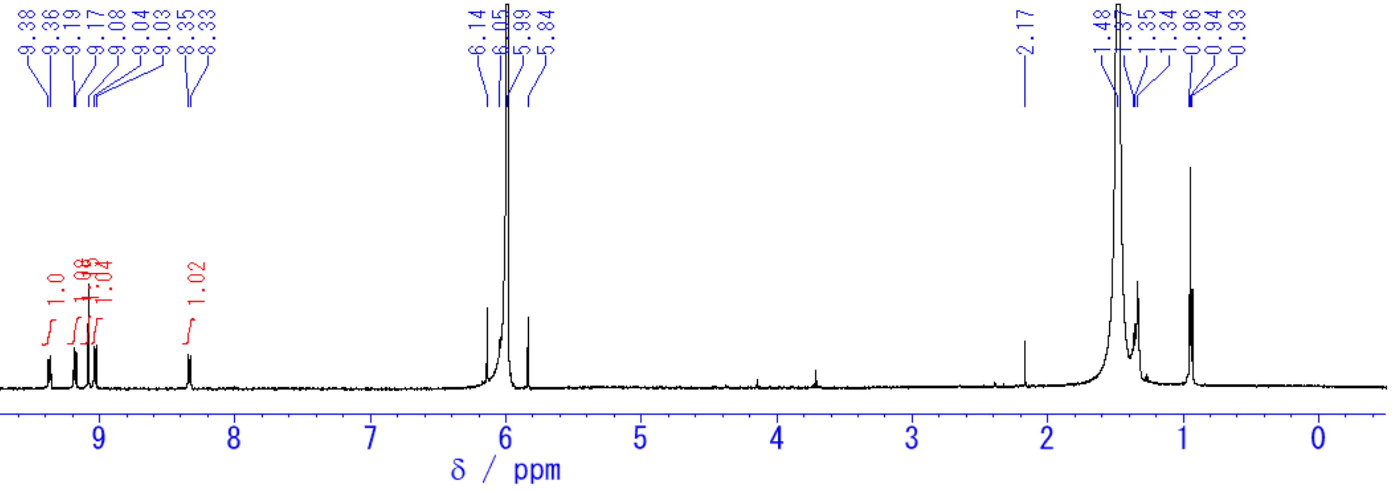

^{1}H (600 MHz) NMR spectrum of **$F_8$7PHN** in 1,1,2,2-tetrachloroethane-d_2 (100 °C).

5. References

S1. Cavalli, L. *Ann. Rep. NMR Spectrosc.,* **1976**, *6B*, 43–222.

S2. Dewar, M. J. S.; Grisdale, P. J. *J. Am. Chem. Soc.,* **1962**, *84*, 3541–3546.

S3. Kelley, C.; Lu, S.; Parhi, A.; Kaul, M.; Pilch, D. S.; LaVoie, E. J. *Eur. J. Med. Chem.*, **2013**, *60*, 395–409.

S4. Shoesmith, J. B.; Rubli, H. *J. Chem. Soc.*, **1927**, 3098–3106.

S5. Jiang, J.; Yuan, D.; Ma, C.; Song, W.; Lin, Y.; Hu, L.; Zhang, Y. *Org. Lett.*, **2021**, *23*, 279–284.

S6. Církva, V.; Jakubík, P.; Strašák, T.; Hrbáč, J.; Sýkora, J.; Císařová, I.; Vacek, J.; Zádný, J.; Storch, J. *J. Org. Chem.,* **2019**, *84*, 1980–1993.

S7. Baycal, A.; Plietker, B. *Eur. J. Org. Chem.*, **2020**, *9,* 1145–1147.

S8. Ravindra, B.; Das, B. G.; Ghorai, P. *Org. Lett.,* **2014**, *16*, 5580–5583.

S9. Okamoto, H.; Takane, T.; Gohda, S.; Kubozono, Y.; Sato, K.; Yamaji, M.; Satake, K. *Chem. Lett*., **2014**, *43*, 994–996.

S10. Okamoto, H.; Yamaji, M.; Gohda, S.; Sato, K.; Sugino, H.; Satake, K. *Res. Chem. Intermed*., **2013**, *39*, 147–159.

S11. Fujino, S.; Yamaji, M.; Okamoto, H.; Mutai, T.; Yoshikawa, I.; Houjou, H.; Tani, F. *Photochem. Photobiol. Sci*., **2017**, *16*, 925–934.

S12. Gaussian 16, Revision C.01, Frisch, M. J.; Trucks, G. W.; Schlegel, H. B.; Scuseria, G. E.; Robb, M. A.; Cheeseman, J. R.; Scalmani, G.; Barone, V.; Petersson, G. A.; Nakatsuji, H.; Li, X.; Caricato, M.; Marenich, A. V.; Bloino, J.; Janesko, B. G.; Gomperts, R.; Mennucci, B.; Hratchian, H. P.; Ortiz, J. V.; Izmaylov, A. F.; Sonnenberg, J. L.; Williams-Young, D.; Ding, F.; Lipparini, F.; Egidi, F.; Goings, J.; Peng, B.; Petrone, A.; Henderson, T.; Ranasinghe, D.; Zakrzewski, V. G.; Gao, J.; Rega, N.; Zheng, G.; Liang, W.; Hada, M.; Ehara, M.; Toyota, K.; Fukuda, R.; Hasegawa, J.; Ishida, M.; Nakajima, T.; Honda, Y.; Kitao, O.; Nakai, H.; Vreven, T.; Throssell, K.; Montgomery, Jr., J. A.;

Peralta, J. E.; Ogliaro, F.; Bearpark, M. J.; Heyd, J. J.; Brothers, E. N.; Kudin, K. N.; Staroverov, V. N.; Keith, T. A.; Kobayashi, R.; Normand, J.; Raghavachari, K.; Rendell, A. P.; Burant, J. C.; Iyengar, S. S.; Tomasi, J.; Cossi, M.; Millam, J. M.; Klene, M.; Adamo, C.; Cammi, R.; Ochterski, J. W.; Martin, R. L.; Morokuma, K.; Farkas, O.; Foresman, J. B.; Fox, D. J. Gaussian, Inc., Wallingford CT, 2019.

S13. Becke, A. D. *J. Chem. Phys*., **1993**, *98* 5648–5652.

S14. Stratmann, R. E.; Scuseria, G. E; Frisch, M. J. *J. Chem. Phys*., **1998**, *109*, 8218–8224.
